# Murine and related chapparvoviruses are nephro-tropic and produce novel accessory proteins in infected kidneys

**DOI:** 10.1371/journal.ppat.1008262

**Published:** 2020-01-23

**Authors:** Quintin Lee, Matthew P. Padula, Natalia Pinello, Simon H. Williams, Matthew B. O'Rourke, Marcilio Jorge Fumagalli, Joseph D. Orkin, Renhua Song, Babak Shaban, Ori Brenner, John E. Pimanda, Wolfgang Weninger, William Marciel de Souza, Amanda D. Melin, Justin J.-L. Wong, Marcus J. Crim, Sébastien Monette, Ben Roediger, Christopher J. Jolly

**Affiliations:** 1 Centenary Institute, Faculty of Medicine and Health, The University of Sydney, Sydney, NSW, Australia; 2 Proteomics Core Facility, University of Technology Sydney, Sydney, NSW, Australia; 3 Center for Infection & Immunity, Mailman School of Public Health, Columbia University, New York, NY, United States of America; 4 Kolling Institute of Medical Research, Faculty of Medicine and Health, The University of Sydney, Sydney, NSW, Australia; 5 Virology Research Center, School of Medicine of Ribeirão Preto of the University of São Paulo, Ribeirão Preto, Brazil; 6 Institut de Biologia Evolutiva, CSIC-Universitat Pompeu Fabra, Barcelona, Spain; 7 Department of Anthropology and Archaeology, University of Calgary, Alberta, Canada; 8 Melbourne Integrative Genomics, University of Melbourne, Melbourne, Victoria, Australia; 9 Department of Veterinary Resources, Weizmann Institute of Science, Rehovot, Israel; 10 Lowy Cancer Research Centre, University of New South Wales Sydney, Sydney, NSW, Australia; 11 Department of Dermatology, Medical University of Vienna, Vienna, Austria; 12 Department of Medical Genetics and Alberta Children’s Hospital Research Institute, Cumming School of Medicine, University of Calgary, Alberta, Canada; 13 Microbiology and Aquatic Diagnostics, IDEXX BioAnalytics, Discovery Drive, Columbia, MO, United States of America; 14 Laboratory of Comparative Pathology, Center of Comparative Medicine and Pathology, Memorial Sloan Kettering Cancer Center, The Rockefeller University, Weill Cornell Medicine, New York, NY, United States of America; 15 Autoimmunity, Transplantation, Inflammation (ATI) Disease Area, Novartis Institutes for Biomedical Research, Basel, Switzerland; University of Kansas Medical Center, UNITED STATES

## Abstract

Mouse kidney parvovirus (MKPV) is a member of the provisional genus Chapparvovirus that causes renal disease in immune-compromised mice, with a disease course reminiscent of polyomavirus-associated nephropathy in immune-suppressed kidney transplant patients. Here we map four major MKPV transcripts, created by alternative splicing, to a common initiator region, and use mass spectrometry to identify “p10” and “p15” as novel chapparvovirus accessory proteins produced in MKPV-infected kidneys. p15 and the splicing-dependent putative accessory protein NS2 are conserved in all near-complete amniote chapparvovirus genomes currently available (from mammals, birds and a reptile). In contrast, p10 may be encoded only by viruses with >60% amino acid identity to MKPV. We show that MKPV is kidney-tropic and that the bat chapparvovirus DrPV-1 and a non-human primate chapparvovirus, CKPV, are also found in the kidneys of their hosts. We propose, therefore, that many mammal chapparvoviruses are likely to be nephrotropic.

## Author summary

Parvoviruses are small, genetically simple single-strand DNA viruses that remain viable outside their hosts for very long periods of time. They cause disease in several domesticated species and in humans. Mouse kidney parvovirus (MKPV) is a causative agent of kidney failure in immune-compromised mice and is the only member of the provisional Chapparvovirus genus for which the complete genome including telomeres is known. Here, we show that MKPV propagates almost exclusively in the kidneys of mice infected naturally, wherein it produces novel accessory proteins whose coding regions are conserved in amniote-associated chapparvovirus sequences. We assemble a closely related complete viral genome present in DNA extracted from the kidney of a wild *Cebus imitator* monkey, and show that another related chapparvovirus is preferentially found in kidneys of the vampire bat *Desmodus rotundus*. We conclude that many mammal-hosted chapparvovirus are adapted to the kidney niche and may therefore cause disease following kidney stress in multiple species.

## Introduction

Parvoviruses are small, non-enveloped, polyhedral, single-strand DNA viruses with genomes 4–6kb in length which bear short (120–600 base) terminal repeats (TRs) that form hairpin telomeres. All parvoviral genomes comprise two major genes encoding a non-structural replication protein NS1 (gene *rep*) and a capsid protein VP (gene *cap*). Alternative splicing or alternative translation initiation sites can allow the production of truncated forms of VP; all sharing the same C-terminal region [1, 2]. Open reading frames (ORFs) usually overlapping the NS1 or VP reading frames encode smaller genus-specific accessory proteins. Parvoviruses can only replicate when the host cell itself replicates. Furthermore, many members of the *Dependoparvovirus* genus (e.g. adeno-associated virus, AAV) can only replicate if a helper virus is also present [1, 3], but this is not a universal feature of *Dependoparvovirus*–close avian relatives of AAV that cause Derzsy’s disease in geese and Muscovy ducks replicate autonomously [4].

Vertical transmission of parvoviruses across the placenta can kill developing embryos or newborns in domesticated species such as dogs and pigs [5, 6], but many parvoviruses are highly adapted to infecting specific cell types. For instance, *Erythroparvovirus* B19 infects red blood cell precursors in humans, potentially inducing anaemia [7], and even though AAV2 can transduce many cell types, it is naturally liver-adapted and targets the liver if intravenously injected [8]. Horizontal transmission of the newly-identified mouse kidney parvovirus (MKPV) induces adult renal failure in severely immune-deficient laboratory mice, without obvious pathology in other tissues [9]. Co-incidentally, a virus very similar to MKPV was identified in mice living wild in New York City (NYC), with greater incidence in adults than juveniles, and dubbed murine chapparvovirus (MuCPV), but the state of kidney disease was not assessed in that study [10]. The *rep* plus *cap* sequence of MuCPV, lacking TRs, was originally assembled from the faecal virome of house mice living wild in New York City (NYC; accession MF175078) [10]. Independently, a full-length 4,442 nt sequence of MKPV, including TRs, was assembled from the kidney transcriptomes of two renal disease-affected immune-deficient *Rag1*^*-/-*^ mice in the colony of the Centenary Institute, Sydney, Australia (CI; accession MH670587), and a 3.5 kb fragment of MKPV encompassing NS1 and VP was then amplified by PCR from the kidneys of immune-deficient mice necropsied at Memorial Sloan Kettering Cancer Center, NYC (MSKCC; accession MH670588) [9]. The MuCPV and MKPV genomes are 98% identical to one another at the nucleotide level; thus, they belong to the same species according to ICTV guidelines.

MKPV and MuCPV are only distantly related to other known murine parvoviruses and are members of the provisional genus Chapparvovirus; so-called because the earliest examples, discovered by metagenomic analyses, were found in chiropteran, avian and porcine hosts (i.e. bats, birds and pigs) [11–13]. Recently, additional amniote chapparvovirus (ChPV) sequences were discovered by screening of draft genome assemblies and presumed to reflect parvoviral infection of the source animal rather than viral genome integration [14]. The growing list of potential hosts now includes marsupials and fish; furthermore, ChPV-derived endogenous viral elements (EVEs) were discovered in some invertebrate genomes [15–17]. This discovery demonstrated that ChPVs are an ancient lineage within the family *Parvoviridae*, and phylogenetic analysis suggested that ChPV form a parvoviral subfamily distinct from the two currently established parvoviral subfamilies *Parvovirinae* and *Densovirinae* [16]. Curiously, extant fish-associated ChPV are more related to ancient invertebrate ChPV-derived EVEs than to extant amniote-associated ChPVs [16].

MKPV is currently the only ChPV sequence that includes terminal repeats (TR), and the only ChPV known to be viable, infective or pathogenic, to date. Here, we extend our characterisation of MKPV and related viruses. Specifically, we assess global distribution of MKPV, analyse MKPV tropism, map the major MKPV transcripts, describe a closely-related full-length ChPV from a primate kidney, and demonstrate the production of novel accessory proteins from MKPV transcripts that are conserved in chapparvovirus genomes. Our data suggest that multiple mammal-associated chapparvovirus species may be adapted to a nephron niche.

## Results

### MKPV and MuCPV share identical telomeres

**Fig 1:**
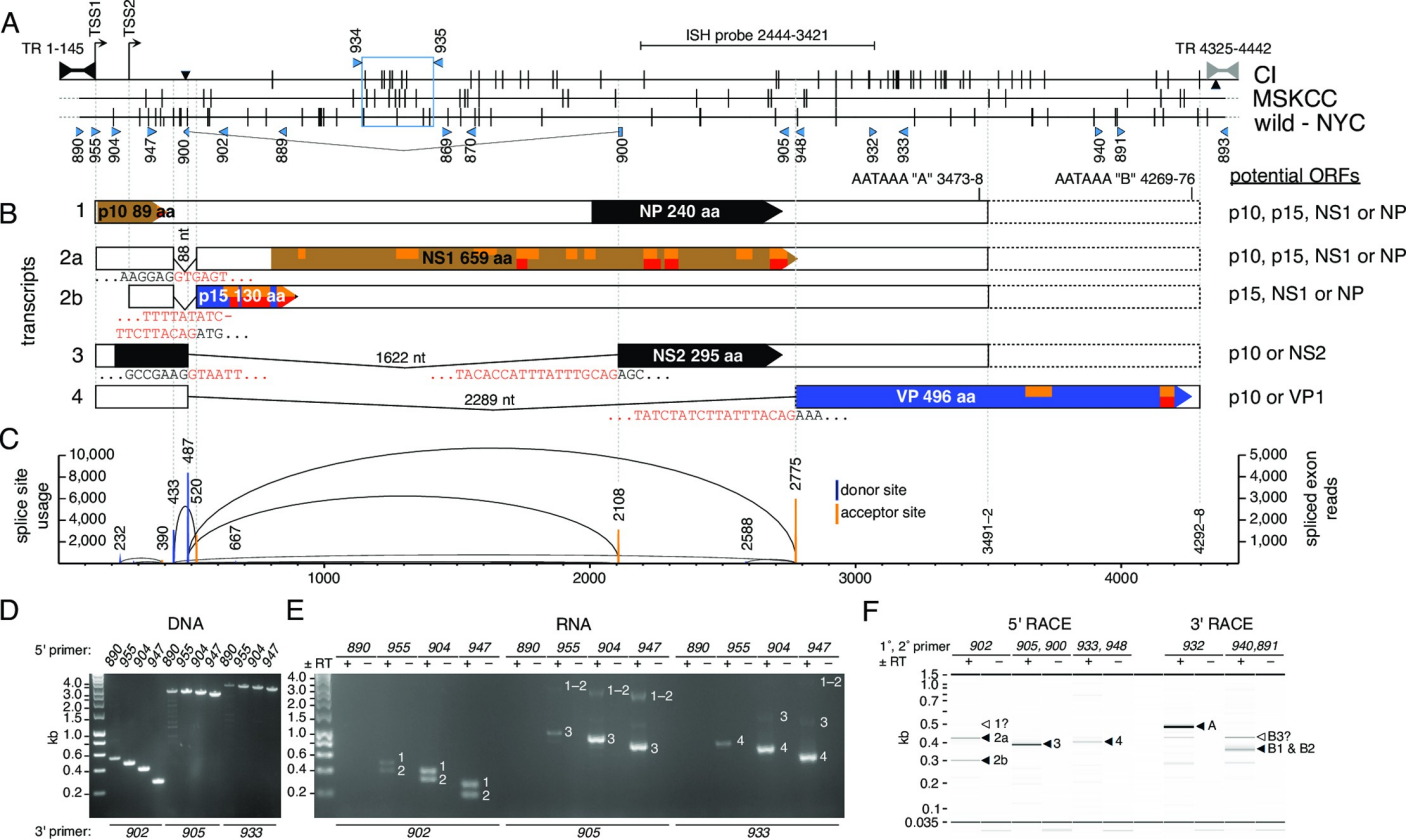
Map of the MKPV genome. **(A-B)** Maps of the MKPV/MuCPV strains from Centenary Institute (CI, accession MH670587), Memorial Sloan Kettering Cancer Center (MSKCC, accession MH670588) and New York City basements (wild-NY, MF175078). “Bowties” indicate terminal repeats (TR). **(A)** Single nucleotide variations (SNV) between the CI, MSKCC and wild-NY accessions. Vertical lines—differences between accessions. Half height vertical lines—polymorphisms within an accession. ▼; 2 bp insertion in the CI strain. ▲; 1 bp insertion in a CI sub-strain. Dashed lines—missing extremities in MSKCC and wild-NY accessions, which consist of the exterior inverted repeats in the full-length CI sequence. **(B-C)** Alternative splicing allows production of the polypeptides p10, p15, NS1, NS2, NP and VP. Black, brown or blue shading indicate the relative reading frames of ORFs. p15, p10 and NP could theoretically be produced from multiple transcripts. Orange or red indicate peptides present in LC-MS/MS datasets PXD014938 (this paper) or PXD010540 [9], respectively. Exon or intron sequences flanking splice sites are shown in black or red text, respectively. **(C)** Quantitation of spliced MKPV reads in RNAseq data pooled from two MKPV-infected kidneys. Columns indicate splice site usage (left y-axis); heights of arcs (right y-axis) indicate the abundance of specific splice combinations. See S4 Table for more information. **(D-E)** Detection of spliced transcripts by RT-dependent PCR, using primers mapped in A-B. Input templates were MKPV-infected **(D)** kidney DNA or **(E)** DNAse/ExoI-treated kidney RNA, converted (+RT) or mock-converted (-RT) to cDNA. RT-PCR products corresponding to transcripts 1 to 4 are indicated by white numbers. **(F)** Mapping of transcription start and stop sites by RACE. See S1 Fig for RACE details. Major 5’ and 3’ RACE products, indicated by black arrows and corresponding to transcripts 2 to 4 or polyadenylation signals A and B, were gel-purified and Sanger sequenced. Other RACE products mentioned in the text are indicated by white arrows.

Using primers 889, 890, 891 and 893 based on the CI-MKPV strain (Fig 1A and S1 Table), the 5’- and 3’-sequences of NYC-MuCPV and MSKCC-MKPV were extended outwards to include the innermost repeat and hairpin region of each TR (see NCBI accessions MF175078.2 and MH670588.2). This confirmed that MuCPV and MKPV share identical heterotelomeric TRs (Fig 1A).

### MKPV expresses “p10” and “p15” accessory proteins

All viable parvoviruses encode NS1 and VP, and production of these proteins in MKPV-infected tissue was confirmed previously by liquid chromatography-tandem mass spectrometry (LC-MS/MS) [9]. However, both MKPV and the extended MuCPV sequence have potential to produce several other polypeptides from ORFs >25 aa in length. We performed a new independent LC-MS/MS analysis of an MKPV-infected kidney and an uninfected kidney, focusing on novel MKPV accessory proteins (dataset PXD014938). In addition, we mined our previous LC-MS/MS datasets (PXD010540) [9] for trypsin-derived peptides predicted by these ORFs. These independent analyses re-identified NS1 and VP, as expected. They also identified twelve peptides (with E-values <0.001) covering 65% of a 14.7 kDa polypeptide “p15” (Fig 1B and S2 and S3 Tables). p15-derived peptides were more abundant in the infected LC-MS/MS data than peptides derived from NS1 or VP (S2 Table). Two peptides covering the C-terminal 16% of a 9.8 kDa polypeptide, “p10”, situated immediately downstream of the left TR were also detected (see Fig 1B and S2 and S3 Tables). No other MKPV-derived peptides were detected in infected kidneys and no MKPV-derived peptides were detectable in extracts from uninfected control kidneys.

### MKPV mRNA splicing

Previous qualitative comparison of Illumina reads with the confirmed MKPV genome indicated the presence of three major MKPV introns (see accession MH670587). We quantified MKPV splicing in the RNAseq data (GSE117710) from two independent MKPV-infected kidneys (S4 Table). This confirmed that the two donor sites and three acceptor sites used by the above-mentioned introns accounted for 96% of all detectable MKPV splicing events, with an additional five donor and two acceptor sites accounting for >99% of the remaining splice events (S4 Table, Fig 1C). To directly confirm the major splicing events, we extracted DNA or RNA from kidneys of MKPV^+ve^ *Rag1*^*–/–*^mice and exposed the RNA to DNase I plus Exo I to destroy MKPV DNA. After reverse transcription (“+RT”) or mock reverse transcription (“-RT”), we amplified MKPV cDNA using antisense primers 902, 905 or 933 paired with primers 890, 955, 904 or 947, which are mapped in Fig 1A and 1B. The sense primers “walked” from the hairpin of the left TR (primer 890) to just upstream of the most 5’ major splice donor site (primer 947). Agarose gel analysis of PCR products (S1 Table and Fig 1D and 1E) confirmed that the RNA template was free of MKPV DNA and detected an MKPV transcript that did not use any of the major splice sites (transcript 1) plus the spliced MKPV transcripts 2–4, illustrated in Fig 1B, as the dominant transcripts. The splicing indicated in Fig 1B for transcripts 2–4 was confirmed by Sanger sequencing; similarly, Sanger sequencing confirmed that transcript 1 contained the 88 nt intron intact. Counting of spliced reads or reads mapped to intron sequence uniquely retained in transcript 1 in the RNAseq data (GSE117710) indicated that transcript 1 accounted for 7–13% of MKPV transcripts (S4 Table). This is likely to be a slight over-estimate because MKPV DNA was a trace contaminant in the source RNA [9]. Furthermore, our analysis does not exclude the possibility that a small minority of transcript 1 mRNAs might splice using splice sites 3’ to primer 902 (i.e. minor donors at nt 667, 2189 and 2588 spliced to the acceptor at 2775 –see S4 Table).

Consistently strong RT-PCR yields using primers 947 and 904 (Fig 1E) suggested that transcription start sites for all major transcripts lie 5’ to primer 904. In contrast, RT-PCR yields were consistently weak with primer 955, despite this primer yielding strong amplification from MKPV DNA (Fig 1D). Primer 890, located in the hairpin of the left TR, produced no detectable products in RT-PCR reactions at all (Fig 1E), despite being able to produce products from the MKPV genome (albeit with relatively low efficiency; Fig 1D). This indicated that MKPV’s major transcripts initiate a short distance downstream of the left TR.

### Mapping of transcription start and polyadenylation sites by RACE

To precisely map the 5’-ends of the major MKPV transcripts, we deployed rapid amplification of cDNA ends (RACE) following SMARTer full-length cDNA synthesis (S1A Fig). Sanger-sequencing of the major 5’ RACE products (Fig 1F and S1B Fig) confirmed that they corresponded to transcripts 2–4 –as labelled in Fig 1F. Two transcription start sites, TSS1 and TSS2, were mapped for transcript 2 (Fig 1A and 1F). TSS1 corresponds to nt 147 with “smearing” to nt 144–146 (transcript 2a), while TSS2 corresponds to nt 267 with “smearing” to nt 266 (transcript 2b). The yield of 5’ RACE products (Fig 1F) suggested that transcripts 2a and 2b accumulate to roughly equal proportions. The transcription starts for transcripts 3 to 4 mapped to precisely the same nucleotides as transcript 2a –i.e. TSS1 (Fig 1B) but not TSS2. All of these results were consistent with the RT-PCR reactions in Fig 1E. Since the interior repeat of MKPV’s left TR immediately abuts TSS1 (Fig 1A), transcription predominantly initiates from very near the 3’-end of the left TR.

To map polyadenylation sites, we deployed 3’ RACE (Fig 1F, S1A Fig and S1C Fig). This mapped a major site of polyadenylation (polyA) to nucleotides 3491–2 (“GA”; the A lying at nt 3492 prevented single-base precision–see S1C Fig), which lie 13–14 nt 3’ to a polyA signal at 3473–78 (AATAAA “A”, Fig 1B). Polyadenylation sites were also mapped to nucleotides 4292–3 and 4297–8 (both “CA”), which lie 18–24 nt 3’ to polyA signal “B” at 4269–76 (S1C Fig and Fig 1B). Another polyadenylation site, lying 20–30 nt from the 3’ end of the MKPV genome may also be used (“B3” in Fig 1F), but we did not attempt to map this site precisely because the 3’ TR is extremely resistant to Sanger sequencing.

PolyA signal B is necessarily used by copies of transcript 4 that produce VP protein, and we presume that polyadenylation signal A is used by most copies of transcripts 1–3, but it is possible that all transcripts use a mix of both A and B polyA signals (Fig 1B). Other minor transcription start or polyadenylation sites were indicated by capillary electrophoresis of RACE products, but their yields were too low to be Sanger sequenced (Fig 1F). For instance, a faint 5’ RACE product was detected that might correspond to transcript 1 (“1” in Fig 1F), because it was about 80 bp larger than the 5’ RACE product corresponding to transcript 2A.

In theory, p10 is encoded in all major transcripts that start from TSS1, but not from transcripts starting at TSS2. NS1 could be translated from transcripts 1, 2a or 2b (Fig 1B). The ATG start codon of the p15 ORF abuts the splice acceptor site of transcripts 2a and 2b in Fig 1A and 1B. Therefore, p15 could also be produced from transcripts 1, 2a or 2b. In addition, these transcripts potentially encode NP, a hypothetical ORF in Type 2 ChPV, but sometimes lacking a conventional start ATG codon in Type 1 ChPV [16, 18]. Transcript 3 encodes a two-exon variant of NP that we previously dubbed NS2 [9]; however, we have not detected any peptides by LC-MS/MS that confirm production of NS2 or NP *in vivo*. Transcript 4 shares the splice donor of transcript 3 and encodes the capsid protein VP (Fig 1B and 1C, S4 Table). Minor variants of transcripts 3 and 4 that used the immediately upstream splice donor at nt 433 were detected at about 9 to 15-fold reduced frequencies (Fig 1C, S4 Table). Major and minor VP-encoding transcripts summed to account for 44–56% of all MKPV transcripts in infected kidneys (Fig 1F, S4 Table), which is consistent with capsid protein comprising the bulk of infective parvoviral particles.

### MKPV RNA is restricted to the kidney

**Fig 2:**
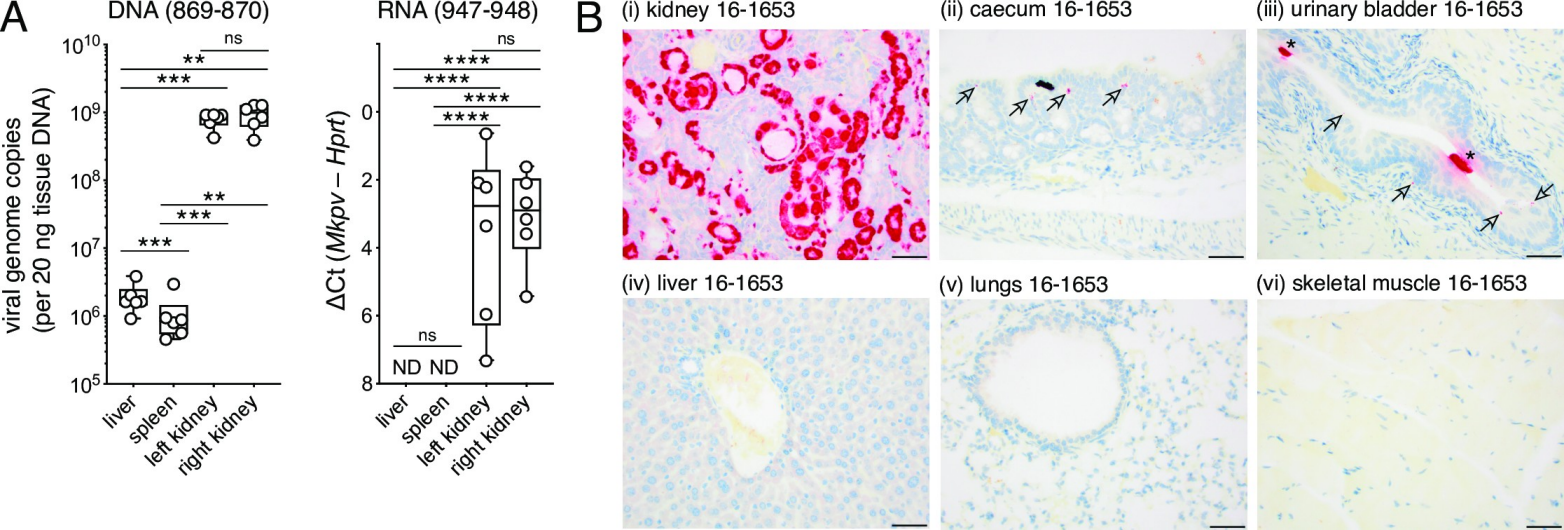
MKPV mRNA is kidney-restricted. **(A)** Relative abundance by qPCR of MKPV genomes (left) or MKPV *cap* mRNA (right) in organs of naturally-infected *Rag1*^*–/–*^mice, using primers 869–870 or 947–948, respectively (Tukey’s box and whisker plots; n = 8). MKPV DNA is presented as viral genome copies. *cap* mRNA abundance is indicated by Ct relative to RT-qPCR for mouse *Hprt* mRNA. ND = not detected. Significance is indicated by asterisks (*, P<0.05; **, P<0.01, ***, P<0.001, ****, P<0.0001; ns, p>0.05; 1-way paired ANOVA with Tukey’s multiple comparisons test). **(B)** ISH for MKPV nucleic acids in necropsy specimens from NSG mouse 16–1653 housed in MSK-WCM in 2016. Scale bar = 25 μm. Arrows in panels (ii-iii) indicate mild multi-focal staining in caecum and urinary bladder; asterisks in panel (iii) indicate casts of necrotic tubular cells sloughed from the kidney into the urinary bladder lumen. Full details of ISH outcomes are listed in S5 Table.

During the natural course of infection, MKPV DNA was detected first in the kidney of young adult mice, then appeared in liver, spleen and blood as infection progressed [9]. The mapping of MKPV RNA splicing (Fig 1) enabled quantitation of MKPV infection *via* qPCR for spliced MKPV RNA in different tissue sites. We extracted DNA and DNA-free RNA from liver, spleen (a proxy for blood) and kidneys of naturally MKPV-infected *Rag1*^*–/–*^mice, then performed qPCR using DNA or cDNA templates. For DNA, we used NS1 primers 869 and 870, as previously reported [9] (Fig 1A). For cDNA, we used primers 947 and 948 (Fig 1A) and a short extension time, which ensured that product formed from spliced transcript 4 and not from MKPV DNA (see Fig 1B). Consistent with our previous study [9], MKPV DNA was much more abundant in kidney than in liver or spleen (Fig 2A). Notably, spliced MKPV RNA was below the detection threshold in liver and spleen, but readily detectable in kidneys (Fig 2A). Because MKPV mRNA was undetectable outside the kidney, we report the difference between Ct for *Hprt* versus MKPV transcripts in Fig 2A, rather than use a spliced cDNA standard curve (unprocessed Ct values are plotted in S3 Fig).

To examine a greater range of tissues, we deployed an MKPV-specific *in situ* hybridization (ISH; RNAscope) probe [9] (Fig 1A) in tissue sections from necropsies of two MKPV^+ve^ NOD-*scid* IL2Rgamma^null^ (NSG) mice. These two mice had histopathologic evidence of chronic inclusion body nephropathy (IBN) and ISH had detected abundant MKPV nucleic acids in tubular epithelial cells [9], as reproduced here (Fig 2Bi). No pathologic change attributed to the virus was observed outside the kidneys on H&E-stained sections. Mild multifocal ISH staining was also observed in the caecum mucosal epithelium and lamina propria of one mouse, but not the other, and in the urinary bladder urothelium (mostly umbrella cells) of both mice (Fig 2Bii-iii, arrows). In addition, there were strongly positive cells in the urinary bladder lumen of both mice, which were presumably casts of necrotic tubular cells sloughed from the kidney (Fig 2BCiii, asterisks). No ISH signal for MKPV was detected in the liver or any of the 20 other tissues screened (Fig 2Biv-vi; S5 Table). Another thirteen tissues sampled during necropsy were not probed because the decalcification process used in their preparation for H&E-staining was incompatible with ISH (see S5 Table).

Together, these data demonstrate that although MKPV can become widely disseminated during the course of infection and may cause latent infection outside the kidney, production of spliced MKPV RNA–which is required for production of new MKPV particles–occurs predominantly or exclusively in the kidneys.

**Fig 3:**
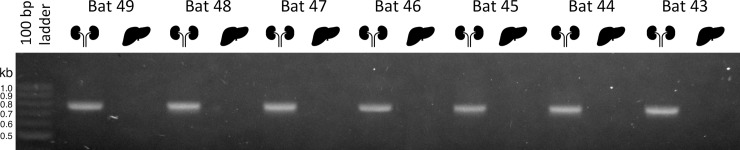
Detection of *rep* DNA of virus DrPV-1 in kidneys and livers of seven *Desmodus rotundus* vampire bats captured in a rural area of Araçatuba city, São Paulo State, Brazil in 2010 [14]. Agarose gel electrophoresis showing the specific 783 bp amplicon. Sample IDs are shown and tissues are indicated by silhouettes.

To test whether viruses related to MKPV might also be kidney-restricted, we screened kidneys and livers from seven vampire bats (*Desmodus rotundus*) previously identified as hosts for the ChPV DrPV-1 [14]. Kidney DNA from all seven vampire bats was re-confirmed as PCR^+ve^ for the DrPV-1 *rep* gene, while liver DNA was uniformly PCR^-ve^ (Fig 3). While this does not prove that DrPV-1 infects kidneys specifically, it demonstrates that DrPV-1 DNA is substantially more abundant in kidneys than livers of host vampire bats.

### MKPV geographical distribution and polymorphism

**Fig 4:**
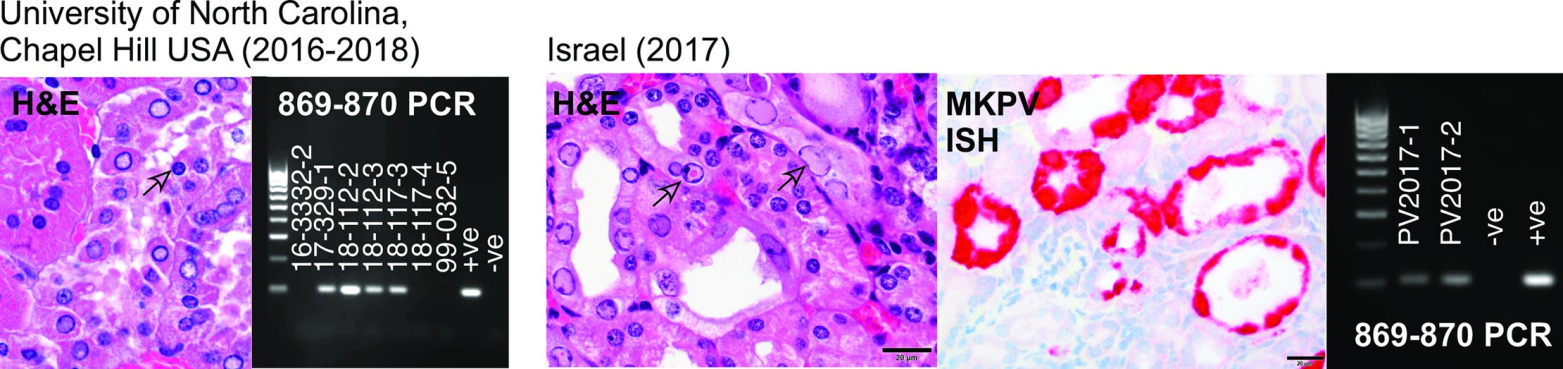
Haematoxylin/eosin (H&E)-staining of historical formalin-fixed paraffin-embedded (FFPE) mouse kidney necropsy samples from the University of North Carolina, Chapel Hill, USA and Israel, paired with agarose gels of 25 cycle 869–870 PCR for MKPV DNA using DNA extracted from FFPE kidney shavings of necropsies from the same sites. For the Israel specimen, ISH for MKPV nucleic acids was also performed. Arrows show examples of inclusion bodies in each H&E stain. PCR panels include size markers at left (0.1–1.0 kb, in 0.1 kb increments) and control DNA at right from MKPV-infected Centenary Institute *Rag1*^*–/–*^mice (+ve) or MKPV-free *Rag1*^*–/–*^mice (-ve, sourced from Australian BioResources, Mittagong NSW).

MKPV/MuCPV was reported in five sites previously: in the wild in NYC, USA and in laboratory mice housed in NYC and Baltimore in the USA, and in laboratory mice from Sydney plus another Australian city [9, 10]. We screened two additional sets of necropsy specimens from laboratory mice with histologically diagnosed IBN by PCR and detected MKPV DNA in laboratory mice housed at University of North Carolina (Chapel Hill, USA) and in Israel (Fig 4). The specimen from Israel was also probed using ISH and abundant MKPV nucleic acids localised to tubular epithelial cells were detected (Fig 4). This increases the number of sites in which MKPV is associated with mouse kidney disease to six sites in three continents.

Alignment of the original CI-MKPV, MSKCC-MKPV and wild NYC MuCPV sequences revealed numerous single nucleotide variations (SNVs) and a two-base insertion in the small intron of CI-MKPV; Sanger and Illumina sequencing data also revealed a few SNVs within each virus strain (Fig 1A). Some of these SNVs were non-synonymous and the resulting changes in amino acid sequences are shown in S2A Fig. Another notable SNV was the insertion of an extra “C” in the right TR of a sub-strain present in one CI mouse, converting the sequence C_4_G_4_ to C_5_G_4_ in the interior repeat (“▲” in Fig 1A), without the insertion of a complementary base in the exterior inverted repeat. This SNV creates an extra 1 nt bubble in the structure predicted to be formed by the right TR (S2B Fig), but whether it results in viable virus remains to be determined.

**Fig 5:**
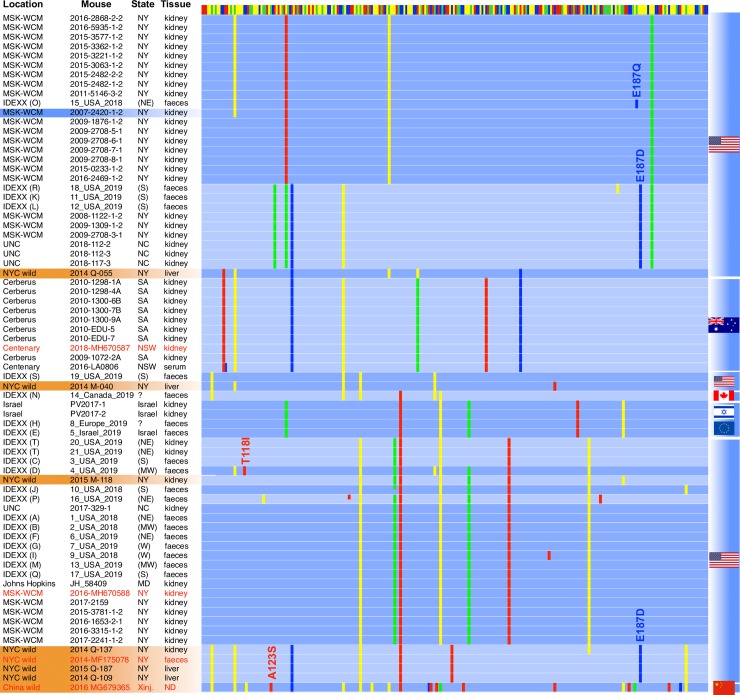
Clustering of MKPV/MuCPV sub-strains based on single nucleotide variations (SNV) in a 267 bp region in NS1 (see blue box in Fig 1A). Data are insufficient to construct a phylogeny. The provenance of each sequence is indicated by text at left and by flags at right, with red text indicating accessions, from top to bottom, MH670587, MH670588, MF175078 and MG679365; for IDEXX BioAnalytics pathology samples, donor institutions are identified by an anonymizing code unique to each institution and by a geographical region where known–each in brackets. Shading over the text indicates infection of a genetically immune-competent strain (blue = laboratory mouse, orange = wild-caught mouse). The coloured bar at the top indicates the consensus sequence (yellow = A, green = G, blue = C, red = T). SNVs varying from the consensus are presented as in Fig 1A, with colour-coding to indicate the non-consensus base. Amino acid changes (five in total) are indicated by “XnnnX”.

Primer pairs were designed to amplify four regions of concentrated polymorphisms in the NS1 and VP ORFs. Only one of these four primer pairs– 934 plus 935 (Fig 1A)–was able to amplify MKPV sequences from historic FFPE samples reliably and this pair was therefore selected for sequencing in a larger sample set. The 934–935 region was amplified from kidney FFPE-specimens or from randomly-selected faecal samples sent to Idexx Laboratories (Columbia, Missouri) from multiple laboratory facilities (in the USA, Canada, Europe and Israel) or from previously-described wild mouse samples from NYC basements [10], then Sanger sequenced from both ends. SNVs were collated in a 267 bp window (the blue box in Fig 1A). Clustering analysis, which incorporated a partial MKPV sequence from *Mus musculus* living wild in Xinjiang, China (accession MG679365), identified 22 MKPV sub-strains, varying by 3–22 SNVs from the consensus sequence; the Xinjiang sequence being the most divergent (Fig 5). The MSK-WCM colonies provided the largest set of time-shifted samples for the same location, from 2007 to present. There was no clear evidence of one strain replacing another over time in the MSK-WCM colonies. Instead, more than one strain was present in the MSK-WCM colonies at most timepoints, and two sub-strains present in MSK-WCM in 2008–2009 and 2015–2017 were identical (within our SNV window) to sub-strains from the University of North Carolina (2018) and Johns Hopkins University (2006), respectively. All of the wild NYC samples shared some SNVs with laboratory strains, mostly located in the same continental region. It was notable that the wild NYC sample Q-055 shared four SNVs with Australian laboratory mice (Fig 5), and the wild NYC sample M-118 shared three SNVs with lab specimens from Europe and Israel (Fig 5). This is consistent with MKPV being carried within immune-deficient lab mice when they were live-exported from the USA to labs outside the Americas, but does not prove it. Virtually all SNVs in the 934–935 window were synonymous, with just four exceptions (Fig 5): Glu187Asp, Glu187Gln, Thr118Ile and Ala123Ser. None of these mutations are likely to affect NS1’s tripartite helicase domain.

### MKPV prevalence

To estimate the prevalence of MKPV in research mice, mouse faecal samples that were submitted to IDEXX BioAnalytics over a seven-month period and representing 78 biomedical research institutions were tested for MKPV by qPCR. Overall prevalence was 5.1%, with 178 positive samples out of 3,517 samples tested. Immune status is unknown for most of the samples. Of those samples designated as representing immunodeficient mice, 16 were positive out of 171 tested (9.4%), and for samples designated as representing immunocompetent mice, 56 out of 513 (10.9%) tested positive for MKPV. It should be noted that many of the faecal samples are likely to represent soiled-bedding sentinel mice, and MKPV prevalence among sentinel mice may differ from colony animals (which may be immunocompetent or immunodeficient) based on a variety of factors including efficiency of transmission by soiled bedding.

Faecal samples from multiple time-points from a single pet shop in Columbia, MO were also submitted to IDEXX BioAnalytics. Seventeen samples were qPCR-positive out of 73 tested (23.3%). In toto, the data establish that MKPV-transmission is common globally in wild, laboratory and pet mice, regardless of immune-deficiency.

### The assembly of an MKPV-related chapparvovirus genome from primate kidney DNA

**Fig 6:**
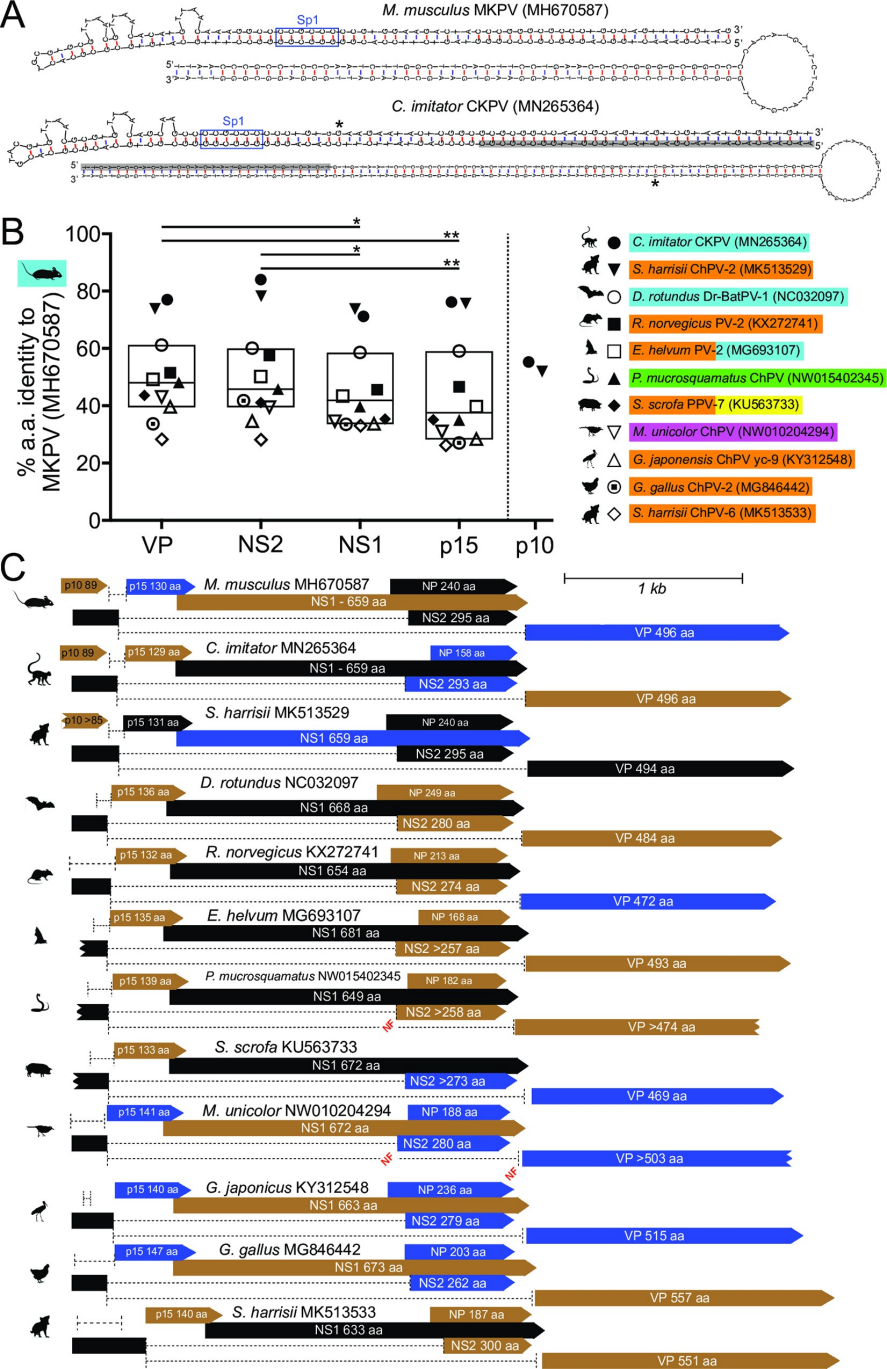
VP, NS1, NS2 and p15 ORFs, introns and TR structures of near-complete amniote ChPV genomes. **(A)** The lowest Gibb’s energy structures predicted for the minus-strand TR regions of MKPV (MH670587) and CKPV (MN265364), with the left TR shown above the right TR. Sp1-binding sequences are boxed in blue. Grey shading in the CKPV TRs indicates sequence recovered from a solitary read, with the 3’-end of the solitary read indicated by “*”. **(B)** The percentage identity at the amino acid level between MKPV ORFs and the corresponding ORFs from nine other near-complete amniote chapparvoviral genomes currently available (MUSCLE alignment [35]). Tukey’s box and whiskers are used. Significant differences in relative ORF conservation (non-parametric Friedman test) are indicated as in the Fig 2 legend. Colours indicate tissue source(s) of the virus sequences: blue–kidney or urine, orange–faeces, yellow–lungs or respiratory tract, green–liver, pink–muscle. **(C)** Maps (same colours and symbols as in Fig 1B) of the near-complete amniote ChPV genomes analysed. Splice sites (or putative splice sites) are detailed in S5B Fig. Ragged ends indicate incomplete ORFs that continue beyond currently available sequence. Genomes sources are: [11, 14, 15, 36–39].

Partial ChPV genomes lacking 5’- or 3’-coding sequences have been assembled from numerous vertebrate species, including from the draft genome of the capuchin monkey (*Cebus capucinus imitator*) [14]. The draft capuchin genome was assembled using DNA extracted from the kidney [19]. We used the genome of MKPV as a scaffold to re-arrange two sequence fragments present in *Cebus imitator* scaffold NW_016109986 into a near-complete ChPV genome, but lacking TRs and with a probable gap in NS1 (S4 Fig). Using SAMtools [20], we mapped high quality reads in the complete capuchin kidney NGS dataset [19] to this draft viral genome, which in-filled a 5 nt gap in the NS1 sequence compared to scaffold NW_016109986.1. By recovering sequences from “soft”-clipped reads at the 5’- and 3’-ends of this new alignment (S4 Fig), we produced a complete ChPV genome with TR lengths longer than MKPV. It should be noted that the first 33 bases and the last 29 bases of the CKPV genome were recovered from a single read each (Fig 6A), but because these reads progressed through unique hairpin regions in the telomeres (the ends of these single reads are indicated by “*” in Fig 6A) they were aligned to the genome extremities non-erroneously. Due to its high level of identity to MKPV (see next paragraph), we dubbed this complete viral genome “capuchin kidney parvovirus” (CKPV, Accession MN265364). The kidney DNA sample used to assemble the CKPV genome (and the draft *Cebus imitator* genome) was extracted in a biological safety cabinet inside a BSL-2 laboratory facility with CL3 protocols to minimise contamination with foreign DNA, but it is no longer available, so we cannot strictly rule out the possibility that CKPV is an extraneous contaminant, nor can we confirm the CKPV genome by PCR or Sanger sequencing.

The CKPV genome is strikingly similar to MKPV (Fig 6) and encodes proteins homologous to MKPV p15 and p10; the VP, NS1, p15 and p10 proteins of CKPV are 77%, 71%, 76% and 55% identical to their MKPV counterparts, respectively (Fig 6B and S5A Fig). Furthermore, the U2-dependent splice donor and acceptor sites used in MKPV for expression of VP and to encode NS2 [9] are conserved in CKPV (Fig 6C and S5B Fig). We predict an NS2 protein in CKPV that is a remarkable 84% identical to MKPV’s NS2 protein (Fig 6B). In contrast, the non-spliced variant of NS2 (i.e. NP) is less conserved because CKPV’s NP ORF is much shorter than MKPV’s (Fig 6C). Finally, the hairpin structures with the lowest Gibbs-free energy predicted for minus strand CKPV TRs are strikingly similar to the structures predicted for minus strand MKPV TRs (Fig 6A).

### ORFs encoding p15 and spliced NS2 are conserved in all amniote chapparvoviruses

Encouraged by ORF conservation between MKPV and CKPV, we searched all near-complete amniote-associated chapparvovirus genomes for conserved ORFs using “Genie” software [21] to identify likely splice donor and acceptor sites for these ORFs. In all cases, we found a p15-like ORF (recently identified independently as ORF-1 [16]) and a 2-exon NS2-like ORF (Fig 6C and S5 Fig). Furthermore, consensus U2-dependent splice donor and acceptor sites were predicted to produce transcripts similar to MKPV transcripts 1–4 in nearly all cases (Fig 6C and S5 Fig). The exceptions (annotated as “NF” for “not found” in Fig 6C) were that the software did not predict splice acceptor sites upstream of the *P*. *mucrosquamatus* or *M*. *unicolor* ChPV VP regions, nor for *M*. *unicolor* ChPV NS2 exon 2. Nonetheless, manual alignment of reading frames implies a functional acceptor site for the *M*. *unicolor* virus NS2 exon 2 and “acceptor-like” sites a short distance upstream of VP in the *P*. *mucrosquamatus*. and *M*. *unicolor* ChPV (see S5 Fig). p15 was significantly less conserved across amniote chapparvoviruses compared to VP and NS2 polypeptides (Fig 6B). On the other hand, VP and NS2 were significantly more conserved than NS1 (Fig 6B).

### The p10 ORF is not present in most chapparvoviruses

Apart from the two closest MKPV relatives, which were found in a capuchin monkey and in faeces of two Tasmanian devils (Fig 6B), an ORF corresponding to p10 was not identifiable in any other ChPV we examined (Fig 6C), even when complete NS2 exon 1 sequence was available (e.g. in DrPV-1). The capuchin CKPV and Tasmanian devil ChPV-2 viruses encode VP, NS2, NS1 and p15 proteins with >71% amino acid identity to the MKPV proteins, but their p10 proteins are <60% identical to MKPV p10 (Fig 6B). Thus, p10 is poorly conserved, or absent in many ChPV sequences, unlike p15 and NS2.

## Discussion

Our data add to the association between MKPV and chronic kidney disease in immune-deficient laboratory mice and demonstrate that MKPV is distributed worldwide. MKPV can be detected in immune-sufficient laboratory mice [9] as well as in wild-living mice in the USA [10] and China, which indicates that MKPV does not require immune-deficiency to propagate and that mice are a natural MKPV host. The relatively low level of SNV diversity in MKPV samples from Australian laboratory mice spanning a decade (Fig 5) suggests that a single laboratory MKPV strain was imported into Australia and has been transmitted horizontally in laboratory mice since, with little or no re-infection from wild mouse sources. In contrast, infection of mouse colonies in the USA by virus from wild mouse pools appears to have occurred repeatedly, but these infections may have occurred prior to the establishment of modern barrier facilities. The MKPV-infected Australian colonies we first reported [9] were all descended from the *Rag1*^tm1Bal^ strain [22]–imported into Australia in about 1994. Fig 5 indicates that this strain is the original source of MKPV in Australian laboratory mice, because all MKPV^+ve^ Australian lab mice seem to carry the same virus strain. Shared SNVs between the Australian MKPV strain and wild mouse sample Q-055 (Fig 5) suggest, but do not prove, that founder infection occurred in the USA. Australian MKPV-free *Rag1*^*–/–*^mice descend from the *Rag1*^tm1Mom^ strain [23] supplied by The Jackson Laboratory (Bar Harbor, Maine, USA). *Mus musculus* is not native to Australia and is thought to have arrived onboard European ships 230 to 420 years ago [24]. Given that other parvoviruses are prevalent in feral house mice in Australia [25] and a virus closely related to MKPV was found in the faeces of a wild scavenger (i.e., a Tasmanian devil, *Sarcophilus harrisii*) in Tasmania [15], it appears likely that MKPV or a related ChPV will be found in mice living wild in Australia. In contrast to *Mus musculus*, *Pseudomys* and other native mice have lived in Australia for four million years or more [26]. Since ChPV2 is only distantly related to the other ChPVs found in *S*. *harrisii* faeces [15], we speculate that *S*. *harrisii* ChPV2 infects rodents that form part of the diet of *S*. *harrisii*, while the other *S*. *harisii*-associated ChPV are marsupial-adapted, because they cluster phylogenetically between avian- and mammalian-associated ChPV [15].

Our initial proteomic analysis of proteins produced by MKPV [9] was limited to polypeptides related to previously predicted ChPV ORFs. Our detection of abundant “p15”-derived peptides and a C-terminal "p10”-derived peptide provides the first direct evidence that ChPVs produce accessory proteins, and do so *in vivo*. p10 has not been predicted by other studies, but p15 corresponds to ChPV ORF-1 which was independently predicted *in silico* during the course of our analyses [16]. We performed the 5’-RACE experiments to test whether transcription initiation site might influence the production of MKPV polypeptides, but with the exception of transcript 2a, all the major MKPV transcripts initiated from the same TSS at or near nt 147 (Fig 1), so the transcription initiation site is not a major determinant of MKPV polypeptide production. The abundance of p15-derived peptides exceeded that of all other MKPV-derived peptides in infected kidneys (S2 Table), surprisingly suggesting that p15 is a major product of MKPV infection. In contrast, we failed to detect any peptides derived from NS2 or NP. Nonetheless, the conservation of splice sites required for NS2 expression in all near-complete amniote ChPV genomes (S5 Fig) combined with detection of major NS2-encoding transcripts in MKPV-infected cells (Fig 1) suggests that NS2 is likely to be a functional ChPV ORF; although it remains plausible that NP is also expressed. We produced consensus protein sequences for p15, NS2 and p10 using Muscle or T-Coffee [27]. As previously noted [16], p15/ORF-1 universally carries clusters of basic amino acids in the C-terminal region suggestive of nuclear localisation signals (see S5A Fig), but searches of Pfam and SwissProt for proteins carrying motifs similar to p15, p10 or NS2/NP on the HMMER server (see Methods) did not find significant matches that could provide any other clues to function. Nonetheless, the so-far universal presence of p15 and NS2 ORFs (or an NS2-like ORF in *Sus scrofa* PPV7 and *Sarcophilus harrisii* ChPV-6 (S5 Fig)) in amniote-associated ChPV indicates that these are likely to be accessory proteins important in the propagation of many or all ChPV. NP and p10 seem to be less conserved. In the case of NP this is due to variation in the position (or absence) of a start ATG codon, although NP might be translated from a non-conventional start codon in some or all instances. In the case of p10, it is due to the absence of any p10-like ORF in most ChPV sequences discovered so far (Fig 6 and S5 Fig).

Comparison of complete primate CKPV and MKPV genomes (Fig 6) demonstrates that chapparvovirus ORFs and TR structures are highly conserved and distinct from the genomes of *Protoparvovirus*, *Ambidensovirus*, *Erythroparvovirus*, *Bocaparvovirus* and *Dependoparvovirus* (reviewed in [1]). Interestingly, the other primate ChPV sequences known so far–the simian parvo-like viruses and *Mafachaviruses* from macaques–cluster with Type 1 porcine ChPVs and not with Type 2 ChPV such as CKPV. Type 1 ChPV nonetheless encode p15 and an NS2-like ORF (this paper and [18, 28]; at the time of writing, *Mafachavirus* sequences were not accessible for inclusion in this paper’s analyses). Precise mapping of the major MKPV transcripts has confirmed the splice donor, splice acceptor and polyadenylation sites originally suggested by RNAseq, quantified usage of major and minor splice sites, and has mapped the dominant transcription start sites to a region immediately 3’ to the left TR. The 5’-ends of MKPV and CKPV are devoid of a consensus TATA-box (i.e. TATAWAW), but each repeat element of the left TR in both MKPV and CKPV encodes an Sp1-binding site. Furthermore, MKPV TSS1 and TSS2 align with the “BBCA^+1^BW” consensus initiator element (S1D Fig; “A^+1”^ indicates the dominant TSS, highlighted in S1D Fig), for which a single mismatch outside the core CA and smearing from the dominant start nucleotide are well tolerated. Similar initiator elements associated with Sp1-binding sites are found in ~30% of human TATA-less promoters [29, 30]. The dominant five MKPV splice sites we have detected (Fig 1C) can account for all MKPV-derived proteins in infected kidneys and we think it unlikely that the remaining splice sites are required for MKPV propagation. Their presence perhaps provides a means for MKPV to rapidly evolve variant proteins or are vestiges of transcriptional profiles of MKPV’s evolutionary precursors (e.g. usage of the 2588:2775 intron alters the C-terminus of NS1, NS2 and NP–see S4 Table). Production of p10 by MKPV might reflect similar adaptive processes.

Detection of MKPV transcripts by qPCR and ISH now demonstrate conclusively that the MKPV strains present in laboratory colonies in Australia and in the USA propagate in kidneys in preference to the liver, intestinal tract and all other soft tissues examined (Fig 2). Furthermore, the absence of any obvious co-occurring virus in MKPV-infected kidneys [9] strongly implies, but does not prove, that MKPV propagates autonomously. We noted previously that MKPV DNA appeared to be more abundant in liver than in other non-kidney sites [9]; furthermore, MuCPV DNA was originally detected in wild mouse livers and anal swabs [10]. Those findings suggested that MKPV/MuCPV might infect liver or the intestinal tract in addition to kidneys. Our current analysis confirmed significantly higher MKPV DNA levels in liver compared to spleen (Fig 2A; adjusted P = 0.0009), but an absence of detectable MKPV RNA in either tissue. This indicates that the liver may act as an MKPV/MuCPV sink or filter during viremia [31], perhaps as a consequence of latent infection, but it is not a site of active MKPV propagation (see Fig 2Biv). The most common source of ChPV sequences to date has been faeces [11–13, 15–18]. Our work shows that MKPV-infection of mouse kidneys leads to the presence of MKPV in faeces *via* shedding in the urine (Fig 2B) and *via* ingestion [9]. Thus, in the absence of data from other tissues, detection of a ChPV sequence in faeces (the most convenient and common specimen used for metagenomics) is not evidence that a ChPV propagates in the intestinal tract. The marked kidney-tropism of MKPV combined with an indication of kidney-tropism for DrPV-1, suggests that viruses closely related to MKPV are adapted to kidney niches in distantly-related mammalian hosts; this may include non-human primate hosts because the CKPV genome was extracted from a capuchin kidney. p10 –encoded by both MKPV and CKPV–does not of itself confer kidney-tropism, because bat DrPV-1 virus lacks a p10 ORF (see Fig 6). Based on studies of AAV [8, 32], it is likely that ChPV VP and/or ChPV promoters determine tropism, and we noted that VP is the most conserved of all amniote ChPV proteins (Fig 5). It is therefore conceivable that VP polymorphisms present in MuCPV compared to MKPV might increase tropism for liver (see [16]). While this paper can’t dismiss that possibility, our data can explain the presence of MuCPV DNA in liver specimens without a requirement for liver tropism.

MKPV infection in immune-deficient *Rag1*^*–/–*^mice shares clinico-pathological features with polyomavirus-associated nephropathy (PVAN), which is a significant complication in immune-suppressed kidney transplant recipients [9, 33]. Our assembly of the complete CKPV genome from the kidney DNA of a capuchin monkey increases the possibility that a pathogenic ChPV might infect human kidneys. Reasoning that urine from immune-suppressed kidney transplant patients is the most likely material in which human ChPV infection might be detected, we mined the fastq files produced by deep-sequencing the urinary DNA of 27 kidney transplant patients ([34], NCBI accession PRJEB28510), searching for ChPV sequences. However, we found none within the datasets. Indeed, we found no parvoviral sequences of any sort within the datasets, but abundant polyomavirus sequences, as originally reported [34]. This limited sample suggests that human kidney ChPV infection might not be widespread–at least not in the USA, but it might be worthwhile nonetheless to determine the occurrence of antibodies against ChPV antigens in human populations. If anti-ChPV antibodies are uncommon in humans then recombinant parvoviral vectors packaged into ChPV capsid might be better able to evade pre-existing antibody-mediated immunity than AAV vectors presently used in the clinic.

## Materials and methods

### Mice and mouse specimens

A colony of naturally MKPV-infected *Cxcr6*^gfp/gfp^ *Rag1*^*–/–*^mice (C57BL/6.Cg) was maintained in the Centenary Institute mouse facility (Sydney, NSW, Australia), as previously described [9]; MKPV-free *Rag1*^*–/–*^mice (C57BL/6.Cg), originally sourced from The Jackson Laboratory, were purchased from Australian BioResources (Moss Vale, NSW, Australia). Multiple colonies representing various strains of mice, including NOD.Cg-*Prkdc*^*scid*^ *Il2rg*^*tm1Wjl*^/SzJ (NSG) and Tac:SW (Swiss Webster) were maintained at Memorial Sloan Kettering Cancer Center (MSK) and Weill Cornell Medicine (WCM), as described previously [9].

Samples analysed for MKPV nucleic acids by PCR or ISH (RNAscope) were formalin-fixed paraffin-embedded (FFPE) specimens archived from historical necropsies of diseased mice from these or other colonies, as described previously [9]. DNA or total RNA was extracted from fresh tissue or FFPE samples using QIAamp DNA Mini Kits or RNeasy Mini Kits from Qiagen (Hilden, Germany), according to the manufacturer’s instructions–with modifications as described previously [9]. Total nucleic acids were extracted from mouse samples submitted to IDEXX BioAnalytics using a commercially available platform (NucleoMag VET Kit; Macherey-Nagel GmbH & Co. KG, Düren, Germany).

### Ethics statement

Fresh tissue specimens were harvested from mice immediately after humane euthanasia with approval for mouse care and experimental procedures by the Animal Welfare Committee, Royal Prince Alfred Hospital (Sydney, NSW, Australia; approval number 2017–043) and in accordance with NSW and Australian Federal legislation and the *Australian code for the care and use of animals for scientific purposes* [40]. Mouse care and experimental procedures were approved by the MSK and WCM Institutional Animal Care and Use Committee (approval number 05-08-017) and maintained in accordance with the National Academy of Sciences’ Guide for the Care and Use of Laboratory Animals in AAALAC International-accredited facilities.

### PCR from MKPV DNA and phylogenetic analysis

All primers mentioned in this report are listed in S1 Table. Most PCRs used 100 ng input DNA, Phire II hot start Mastermix (Thermo Fisher, Vilnius, Lithuania) and 0.5 μM primer pairs. Cycling conditions were as follows: initial denaturation at 98°C for 30 s, followed by 30 cycles (Fig 1D and Fig 5) or 25 cycles (Fig 4) of denaturation at 98°C for 5 s, annealing at 58°C (Fig 1D and Fig 5) or 48˚C (Fig 4) for 5 s, and extension at 72°C for 15 s, and concluded with a final extension at 72°C for 5 min. 5 or 7 μL of the completed PCR product was then loaded onto 1.5% agarose (Vivantis, Selangor Darul Ehsan, Malaysia) gels prepared in 1X TAE buffer (Invitrogen, Grand Island, NY, USA) and 1:10,000 dilution of GelRed (Biotium, Fremont, CA, USA). Electrophoresis was conducted at 110 mA for 60 min before imaging with G:BOX (SynGene, Cambridge, UK) using Syngene’s GeneSnap v7.05 software. PCR amplifications of sequences missing from the 5’- and 3’ends of the previously-described NYC and MSKCC strains of MuCPV/MKPV used primers 890 and 889 for the 5’end or 891 and 893 for the 3’-end, using 30 to 35 cycles of PCR as above. The products were then Sanger-sequenced (Macrogen, Seoul, South Korea).

PCR amplifications for MKPV SNVs used primers 934 and 935 (S1 Table) and were mostly perfomed using Phire II hotstart Mastermix as described above, with the following exceptions. PCR for SNVs at IDEXX BioAnalytics used LA Taq^™^ (TaKaRa Bio, Ōtsu, Japan) and 20 μM primer pairs. Cycling conditions were as follows: initial denaturation at 94°C for 1 min, followed by 40 cycles of denaturation at 94°C for 30 s, annealing at 58°C for 30 s, and extension at 72°C for 30 s, and concluded with a final extension at 72°C for 5 min. PCR for SNVs from wild NYC mice used AmpliTaq Gold 360 Master Mix (Applied Biosystems, Foster City, CA), 50 μM primers and cycling conditions as follows: initial denaturation for 95°C for 8 min, followed by 10 cycles of denaturation at 95°C for 30 s, annealing at 60°C (decreasing by 0.5°C per cycle) for 30 s, and extension at 72°C for 30 s, then a further 35 cycles with similar conditions aside from an annealing temperature of 55°, and concluded with a final extension at 72°C for 7 min. Both strands of PCR products were Sanger sequenced at Macrogen (S Korea; Australian specimens), or at Genewiz Inc. (South Plainfield, NJ; IDEXX and wild NYC specimens). For clustering analysis, nucleotide sequences were aligned in Geneious 10.2.3 [41], and exported to MEGA6 [42] where model selection was performed. A maximum likelihood tree was constructed using the Tamura 3-parameter model [43] with 1000 bootstrap repetitions. The newick tree was exported to FigTree (v1.2.2, http://tree.bio.ed.ac.uk/software/figtree/) for annotation.

### Testing for prevalence of MKPV DNA by IDEXX BioAnalytics

MKPV detection by IDEXX BioAnalytics used a real-time PCR assay based on the IDEXX Laboratories, Inc. proprietary service platform. The MKPV real-time PCR primers and hydrolysis probe were designed with PrimerExpress version 3.0 (Applied BioAnalytics^™^; Waltham, MA, USA) using the genome sequence available in GenBank. The assay was designed and validated to detect 1–10 template copies. Analysis was performed at IDEXX BioAnalytics (Columbia, MO, USA) with standard primer and probe concentrations using the master mix LightCycler 480 Probes Master (Roche Applied Science, Indianapolis, IN, USA) in a commercially available instrument (LightCycler 480; Roche Applied Science). In addition to positive and negative assay controls, a hydrolysis probe-based real-time PCR assay targeting universal prokaryotic (16s rRNA) and eukaryotic reference genes (18s rRNA) was amplified for all samples to confirm the presence of amplifiable DNA and absence of PCR inhibition.

### PCR, RACE and qPCR from MKPV RNA

For splice site confirmations, MKPV-infected kidney RNA was treated or mock-treated with Turbo DNase (Thermo Fisher, 0.4 U/μg RNA) and ExoI (New England Biolabs, 2 U/μg RNA) for 30 min at 37˚C, followed by incubation with DNase Inactivation Reagent (Thermo Fisher, 0.2ul/ μg RNA). First-strand cDNA was produced using random hexamer (Bioline, London, UK) priming and SuperScript III reverse transcriptase (Thermo Fisher, Carlsbad, CA, USA) according to the manufacturer’s protocol. PCR was then performed using input cDNA equivalent to 4 ng RNA and Phire II hot start Mastermix (Thermo Fisher, Vilnius, Lithuania), exactly as described for Fig 1D in the preceding section.

Rapid amplification of cDNA ends (RACE) was performed using reagents from an In-Fusion SMARTer Directional cDNA Library Construction Kit (TaKaRa) and custom primers. First strand cDNA synthesis was performed using the SMARTer CDS primer (S1 Table), SMARTScribe reverse transcriptase (RT) and 500 ng of MKPV-infected kidney RNA, according to the kit instructions. A switch to using a “Template Switch” oligonucleotide (S1 Table) as template occurred during first strand synthesis when the RT incorporated untemplated dCMP nucleotides after encountering the 5’ end of an mRNA template (see S1 Fig). For 5’-RACE, a 2 μL aliquot of the first strand cDNA diluted 1:50 was amplified using Phire II hot start Mastermix (ThermoFisher, Vilnius, Lithuania) and 0.12 μM of 5’-RACE primer (S1 Table) paired with an MKPV-specific antisense oligodeoxynucleotide as return primer. For MKPV transcripts 2a and 2b, primary 5’-RACE with MKPV primer 902 produced two dominant products after 34 PCR cycles (see Fig 1F). For transcripts 3 or 4, primary 5’-RACE used MKPV-specific return primers 905 or 933, respectively, for 25 PCR cycles; a 2 μL aliquot of primary PCR reaction diluted 1:100 was then used as template for semi-nested secondary 5’-RACE using MKPV-specific return primers 900 or 948, respectively, for 34 PCR cycles to produce a single dominant product in each case (see Fig 1F). For 3’-RACE, a 2 μL aliquot of the first strand cDNA diluted 1:50 was amplified in a similar way, using an MKPV-specific sense oligodeoxynucleotide paired with the 3’ RACE primer (S1 Table) as return primer. For polyadenylation site A, primary 3’-RACE used MKPV-specific primer 932 and produced a dominant product after 34 PCR cycles (see Fig 1F). For polyadenylation site B, primary 3’-RACE was performed using MKPV-specific primer 940 for 25 cycles; a 2 μL aliquot of the primary 3’-RACE reaction diluted 1:100 was then used as template for semi-nested 3’RACE using MKPV-specific primer 891 for 34 PCR cycles to produce two or three dominant products (see Fig 1F). Sizes and yields of RACE products (2 μL aliquots) were determined using a Fragment Analyzer (Advanced Analytics; now Agilent, Santa Clara USA) equipped with 55 cm electrophoresis capillaries and reagents capable of resolving dsDNA fragments between 35 and 1500 bp (see Fig 1F), according to the manufacturer’s procedures. Signal traces from each capillary were converted into pseudo-gel images using PROSize 2.0 software (Agilent Technologies, Santa Clara, CA, USA–see Fig 1F). The remaining bulk of the RACE reactions were then resolved by conventional 1.5% agarose gel electrophoresis, as described [9]. DNA bands stained with GelRed (Biotium, Freemont CA, USA) were excised under blue light. The DNA was extracted from the excised agarose using a PCR product purification kit (Promega, Madison, Wisconsin, USA), following the manufacturer’s instructions for agarose-embedded DNA, then Sanger-sequenced (Macrogen, Seoul, S Korea) using the appropriate MKPV-specific primer.

qPCR for MKPV DNA was performed exactly as described before [9], using primers 869 and 870, an annealing temperature of 48˚C and an extension time of 0.5 min. For RNA qPCR, RNA equivalent to 1 ug tissue was pre-treated with DNase, as described above, then reverse-transcribed using oligo-dT primer and SuperScript III reverse transcriptase (ThermoFisher, Carlsbad, CA, USA) at 50 ˚C for 60 min. First-strand cDNA equivalent to 40 ng tissue was then used as template for qPCR reactions using primers 947 and 948, iQ SYBR Green Supermix (Bio-Rad, Hercules, CA, USA), an annealing temperature of 68.5˚C and an extension time of 0.5 min.

### *Cebus imitator* genome assembly

The *Cebus capucinus imitator* genome was assembled as described [19]. In brief, DNA for shotgun sequencing was derived from the kidney of an adult male (id no. Cc_AM_T3) that was killed by a vehicle in Costa Rica. Samples were transported to the laboratory of A.D.M. at Washington University in St Louis, MO under CITES export permit 2015-CR1258/SJ (no. S 1320). Total sequence genome input coverage on the Illumina HiSeq 2500 instrument was approximately 81x (50x fragments, 26x 3kbs, and 5x 8kbs) using a genome size estimate of 3.0Gb. The combined sequence reads were assembled using ALLPATHS-LG software [44] to produce assembly GCA_001604975.1.

### *Desmodus rotundus* specimens and DrPV-1 PCR

DNA was extracted from kidney and liver samples previously obtained from seven DrPV-1^+ve^ *Desmodus rotundus* individuals (vampire bats) captured in a rural area of Araçatuba city, São Paulo State, Brazil, in June 2010 [14] using a QIAamp DNA Mini Kit (Qiagen, USA). Sample collection and handling procedures were approved by the Brazilian Committee on Animal Experimentation (protocol number 00858–2012) and Chico Mendes Institute for the Conservation of Biodiversity; protocol numbers 12.751-3/2009 and 27.346-1/2011. DNA was screened for DrPV-1 sequences using Platinum Taq DNA polymerase and high fidelity PCR buffer (Invitrogen), primers Chap-DRPv-fwd and Chap-DRPv-rev, an initial temperature of 94˚C for 0.5 min, and 35 cycles of 94˚C for 0.25 min, 57˚C for 0.5 min and 68˚C for 1 min. Products were resolved by agarose gel electrophoresis.

### Tissue staining

Tissue sections were prepared and stained with haematoxylin and eosin (H&E) or with an ISH (RNAscope) probe specific for MKPV nucleic acids and positive and negative control probes as previously described [9]. Positive results on caecum and urinary bladder were confirmed by duplicate staining on two serial sections performed in two independent staining runs.

### LC-MS/MS

Kidney protein extracts from an age- and gender-matched pair of MKPV-infected and uninfected *Rag1*^*–/–*^mice were prepared and digested with trypsin as described [9]. Using an Acquity M-class nanoLC system (Waters, USA), 5 μL of each sample was loaded at 15μL/min for 3 minutes onto a nanoEase Symmetry C18 trapping column (180 μm x 20 mm) before being washed onto a PicoFrit column (75 μmID x 300 mm; New Objective, Woburn, MA) packed with Magic C18AQ resin (3 μm, Michrom Bioresources, Auburn, CA). Peptides were eluted from the column and into the source of a Q Exactive Plus mass spectrometer (Thermo Scientific) using the following program: 5–30% MS buffer B (98% Acetonitrile + 0.2% Formic Acid) over 90 minutes, 30–80% MS buffer B over 3 minutes, 80% MS buffer B for 2 minutes, 80–5% for 3 min. The eluting peptides were ionised at 2400V. A Data Dependant MS/MS (dd-MS^2^) experiment was performed, with a survey scan of 350–1500 Da performed at 70,000 resolution for peptides of charge state 2+ or higher with an AGC target of 3e6 and maximum Injection Time of 50ms. The Top 12 peptides were selected fragmented in the HCD cell using an isolation window of 1.4 m/z, an AGC target of 1e5 and maximum injection time of 100ms. Fragments were scanned in the Orbitrap analyser at 17,500 resolution and the product ion fragment masses measured over a mass range of 120–2000 Da. The mass of the precursor peptide was then excluded for 30 seconds.

The MS/MS data files were searched using Peaks Studio X against a database comprised of the *Mus musculus* proteome (UniProt UP000000589) plus all MKPV ORFs >25 amino acids plus a database of common contaminants; with the following parameter settings. Fixed modifications: none. Variable modifications: propionamide, oxidised methionine, deamidated asparagine. Enzyme: semi-trypsin. Number of allowed missed cleavages: 3. Peptide mass tolerance: 10 ppm. MS/MS mass tolerance: 0.05 Da. The results of the search were then filtered to include peptides with a–log_10_P score that was determined by the False Discovery Rate (FDR) of <1%, the score being that where decoy database search matches were <1% of the total matches.

### Bioinformatics and statistics

Following the removal of all reads with hard (H) or soft (S) clipping in the CIGAR string using awk and samtools commands, the SGSeq R package was used to analyse splicing events within MKPV RNAseq data in BAM format [45].

Potential splice donor and acceptor sites in non-MKPV chapparvoviral genomes were sought using “Genie” software [21] online (http://www.fruitfly.org/seq_tools/splice.html). Alignments of chapparvoviral proteins p15, p10, NS1, NS2, NP and VP were performed by MUSCLE [35] or T-Coffee [27], as stated in the text, using MacVector v16 software (MacVector, Inc, North Carolina, USA). Proteins with motifs and domains similar to p10, NS2 and p15 were sought using profile hidden Markov models deployed by the HMMER server (https://www.ebi.ac.uk/Tools/hmmer/). Fastq sequences in NCBI accession PRJEB28510 were downloaded to a local database and searched for reads with any degree of identity to MKPV using MacVector v16 software.

1-way ANOVA (with multiple comparisons tests specified in figure legends) were performed using Prism 7 software (Graphpad Software, Inc).

## Supporting information

S1 Fig (A) Overview of RACE procedure. (B–C) Sanger sequence traces for major products from (B) 5’-RACE or (C) 3’-RACE. In (B), black underlining indicates a BBCA^+1^BW initiator consensus [30], with the dominant initiator nucleotide highlighted.
(PDF)

S2 Fig (A) Comparison of the amino acid sequences of the ORFs encoded by MKPV/MuCPV accessions MH670587, MH670588 and MF175078, in that order. (B) A variant telomere structure created by a single-base insertion in one of the right TR repeats of a sub-strain of CI-MKPV (indicated by “▲” in Fig 1A).
(PDF)

S3 Fig RT-qPCR Ct values for (left) mouse *Hprt* and (right) MKPV *cap* cDNAs scatter-plotted over Tukey’s box and whisker plots. Dashed lines indicate the detection limit by plotting mean Ct values for templates with no reverse-transcription (i.e. no specific template present). ND = no Ct above detection limit.
(PDF)

S4 Fig Pipeline for assembly of a full-length CKPV genome sequence. (PDF)

S5 Fig (A) Alignments of ChPV polypeptides used to calculate the percentage identities plotted in Fig 6B. Perfect or imperfect conservation are indicated by dark or light shading, respectively. The amino acid spanning the exon:exon junction in NS2 polypeptides is boxed in red. (B) Introns mapped in MKPV by RT-PCR, and hypothetical introns in related ChPV species; shown red. Poly-pyrimidine tracts are underlined (with percentage pyrimidines underneath). Start ATG codons are shown in bold; STOP codons for NS1 are boxed. The splice site score generated by Genie software [21] is shown above each actual (MKPV) or hypothetical (all others) splice donor and acceptor site; NS = no score generated.
(PDF)

S1 Table (A) MKPV PCR primers used in this study and (B) product sizes from the MKPV genome or MKPV transcript 1–4 cDNAs.
(PDF)

S2 Table Summary of significant MKPV peptides in LC-MS/MS dataset PXD014938. (PDF)

S3 Table Summary of significant MKPV peptides in LC-MS/MS dataset PXD010540. (PDF)

S4 Table Summary of MKPV splicing in dataset GSE117710. (PDF)

S5 Table Summary of ISH in multiple tissues. (PDF)
