# Supplementary figures and images for: Murine and related chapparvoviruses are nephro-tropic and produce novel accessory proteins in infected kidneys
Source: PLoS Pathog. 2020 Jan 23;16(1):e1008262. doi: 10.1371/journal.ppat.1008262 (PMC6999912; doi:10.1371/journal.ppat.1008262)

(A) 5' and 3'  
RACE overview

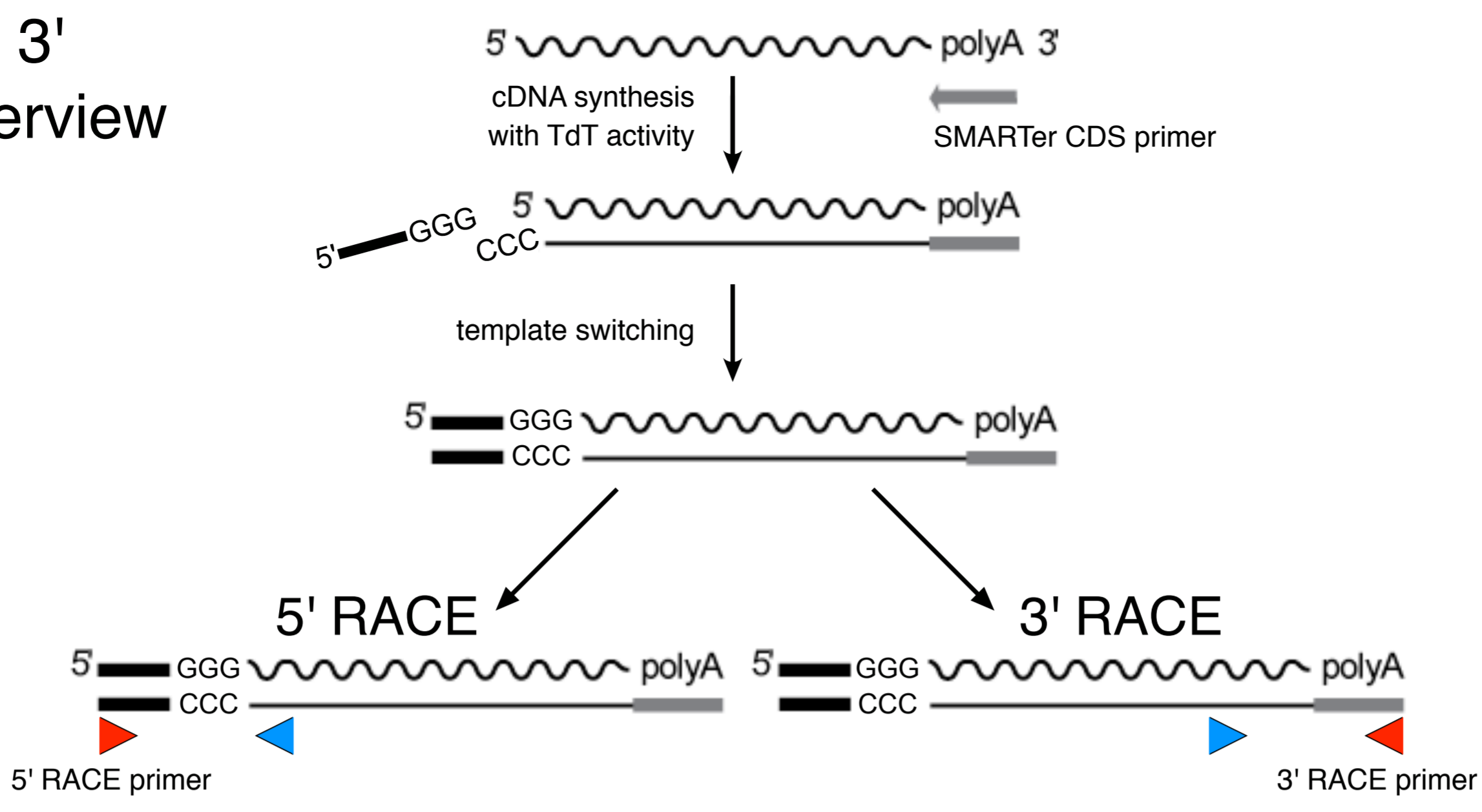

(B) 5' RACE

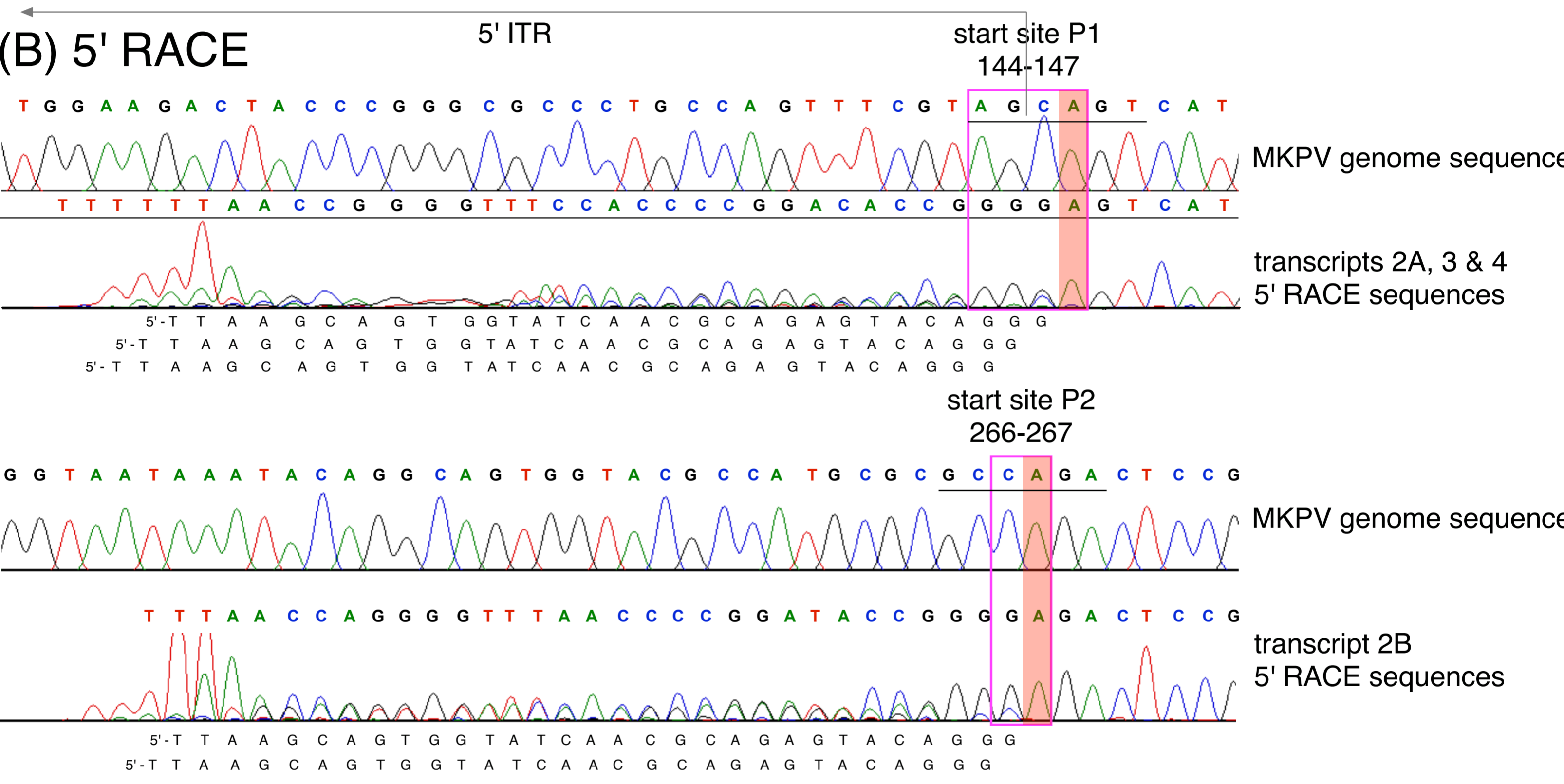

(C) 3' RACE

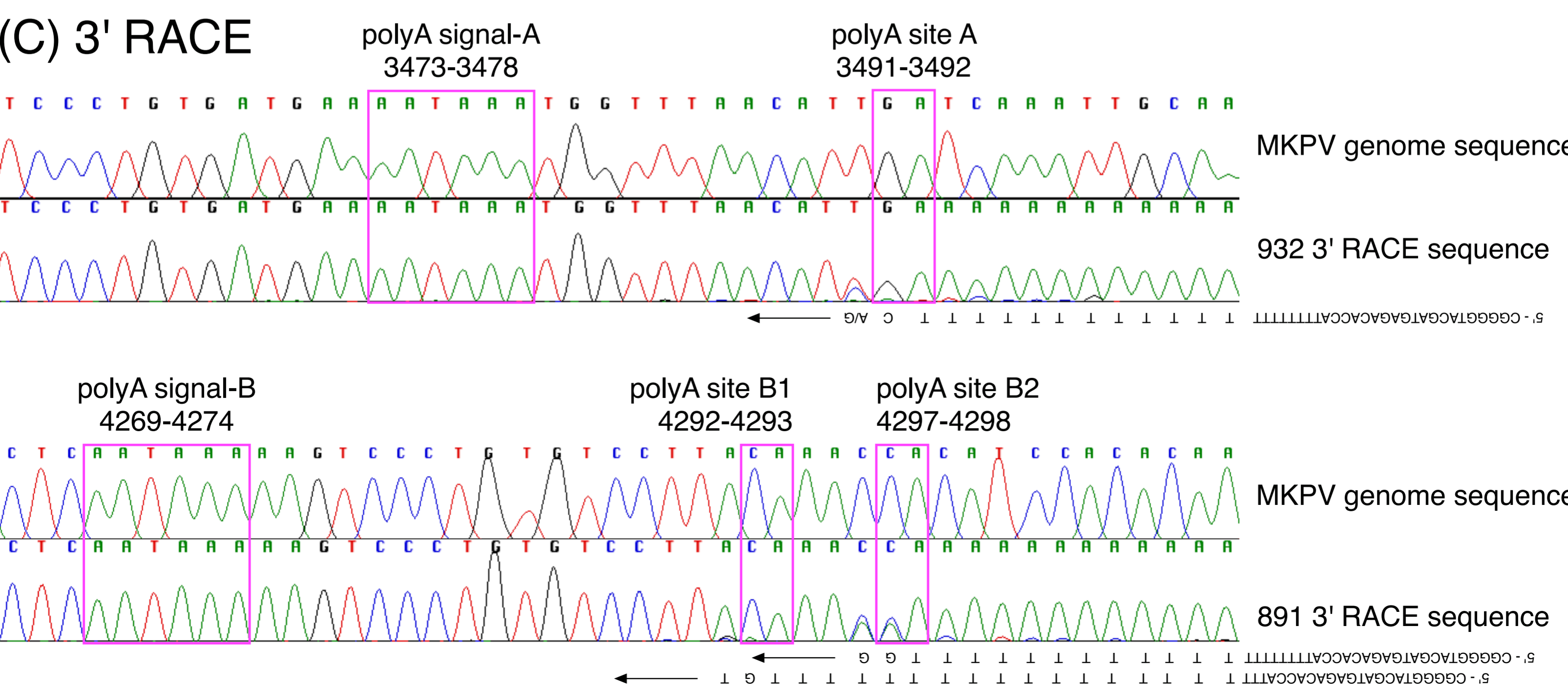

Supplement: S1 Fig — (A) Overview of RACE procedure. (B–C) Sanger sequence traces for major products from (B) 5’-RACE or (C) 3’-RACE. In (B), black underlining indicates a BBCA+1BW initiator consensus [30], with the dominant initiator nucleotide highlighted. (PDF) [file ppat.1008262.s001.pdf]

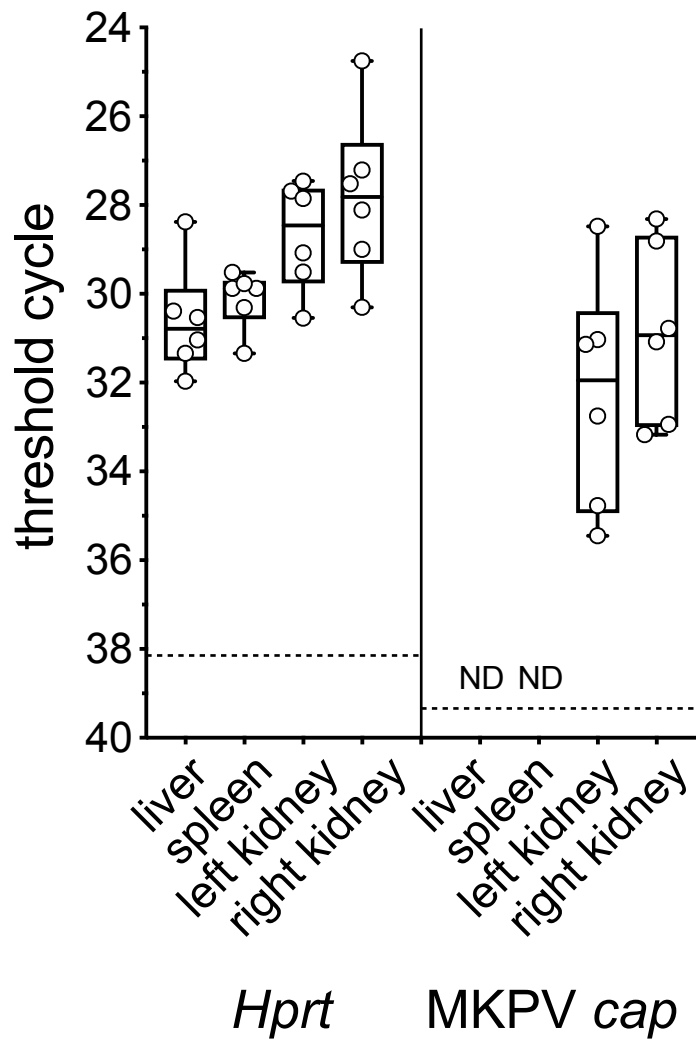

Supplement: S3 Fig — RT-qPCR Ct values for (left) mouse Hprt and (right) MKPV cap cDNAs scatter-plotted over Tukey’s box and whisker plots. Dashed lines indicate the detection limit by plotting mean Ct values for templates with no reverse-transcription (i.e. no specific template present). ND = no Ct above detection limit. (PDF) [file ppat.1008262.s003.pdf]
