# Supplementary material for: Murine and related chapparvoviruses are nephro-tropic and produce novel accessory proteins in infected kidneys
Source: PLoS Pathog. 2020 Jan 23;16(1):e1008262. doi: 10.1371/journal.ppat.1008262 (PMC6999912; doi:10.1371/journal.ppat.1008262)
Supplement: S4 Fig — (PDF) [file ppat.1008262.s004.pdf]

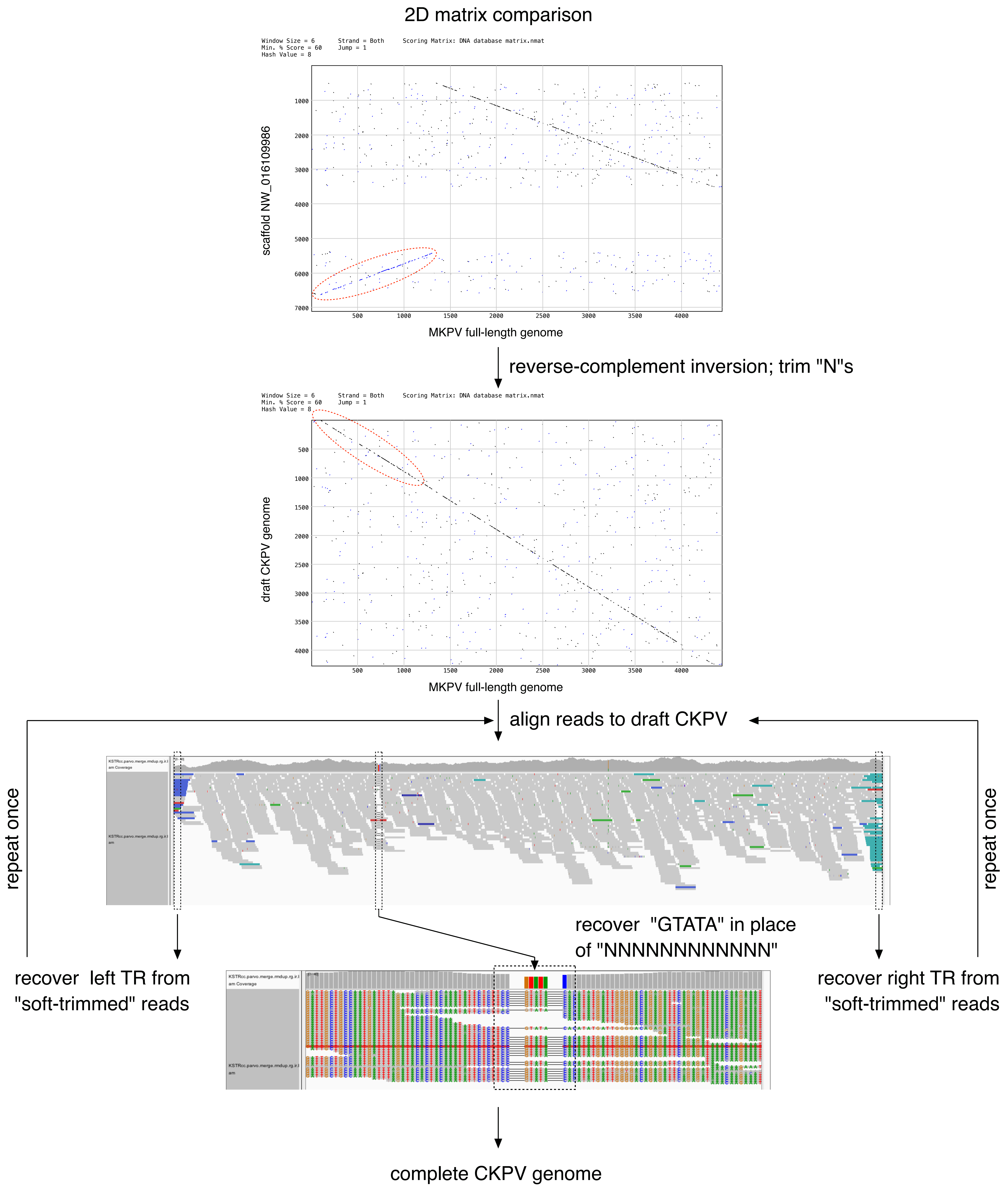
2D matrix comparison
Window Size = 6 Strand = Both Scoring Matrix: DNA database matrix.nmat
Min. % Score = 60 Jump = 1
Hash Value = 8
scaffold NW_016109986
MKPV full-length genome
reverse-complement inversion; trim "N"s
Window Size = 6 Strand = Both Scoring Matrix: DNA database matrix.nmat
Min. % Score = 60 Jump = 1
Hash Value = 8
draft CKPV genome
MKPV full-length genome
align reads to draft CKPV
repeat once
repeat once
recover "GTATA" in place of "NNNNNNNNNNNN"
recover left TR from "soft-trimmed" reads
recover right TR from "soft-trimmed" reads
complete CKPV genome
