# Supplementary material for: Murine and related chapparvoviruses are nephro-tropic and produce novel accessory proteins in infected kidneys
Source: PLoS Pathog. 2020 Jan 23;16(1):e1008262. doi: 10.1371/journal.ppat.1008262 (PMC6999912; doi:10.1371/journal.ppat.1008262)
Supplement: S5 Fig — (A) Alignments of ChPV polypeptides used to calculate the percentage identities plotted in Fig 6B. Perfect or imperfect conservation are indicated by dark or light shading, respectively. The amino acid spanning the exon:exon junction in NS2 polypeptides is boxed in red. (B) Introns mapped in MKPV by RT-PCR, and hypothetical introns in related ChPV species; shown red. Poly-pyrimidine tracts are underlined (with percentage pyrimidines underneath). Start ATG codons are shown in bold; STOP codons for NS1 are boxed. The splice site score generated by Genie software [21] is shown above each actual (MKPV) or hypothetical (all others) splice donor and acceptor site; NS = no score generated. (PDF) [file ppat.1008262.s005.pdf]

Figure S5A

VP

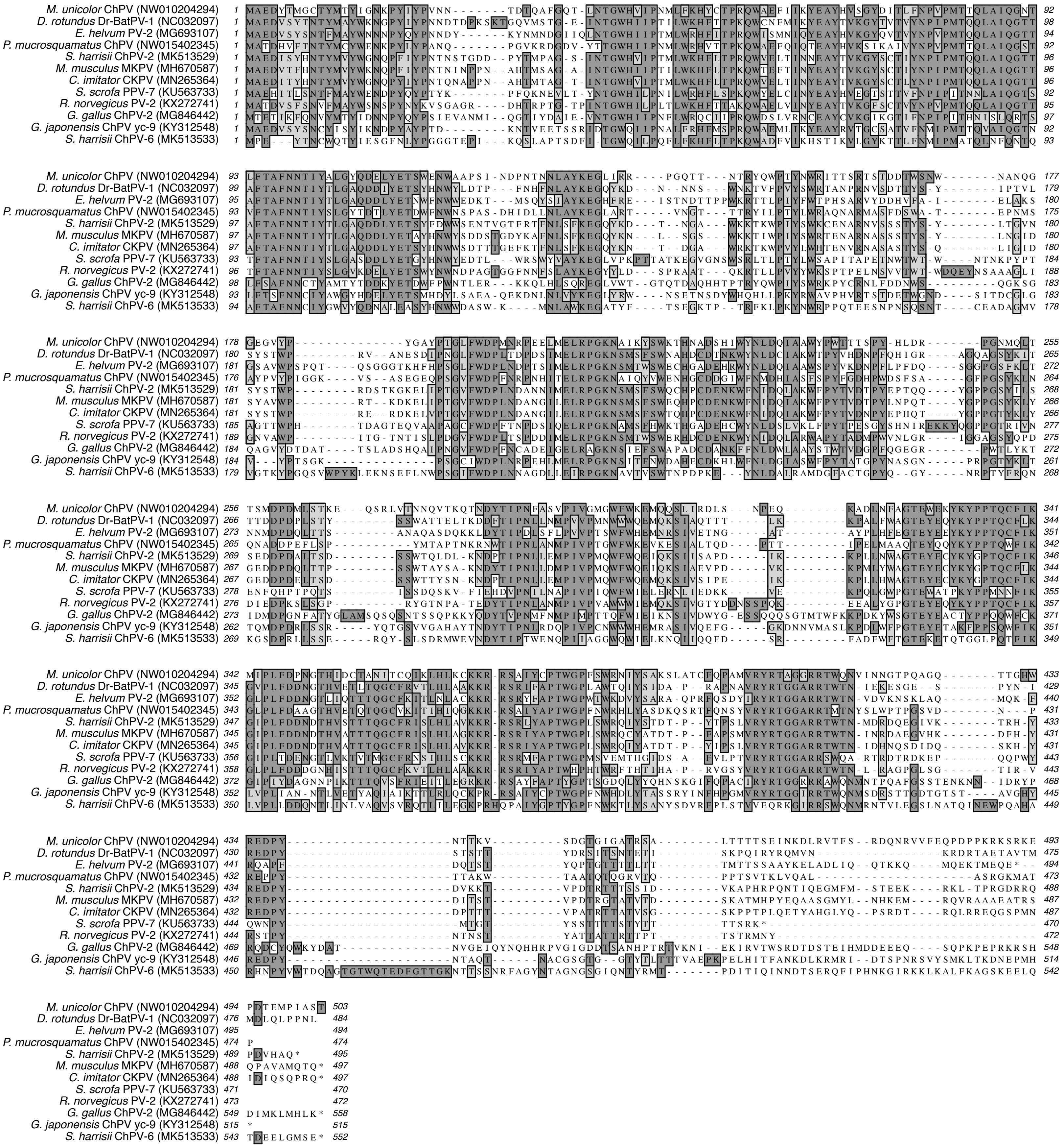

Figure S5A (cont.)

NS2

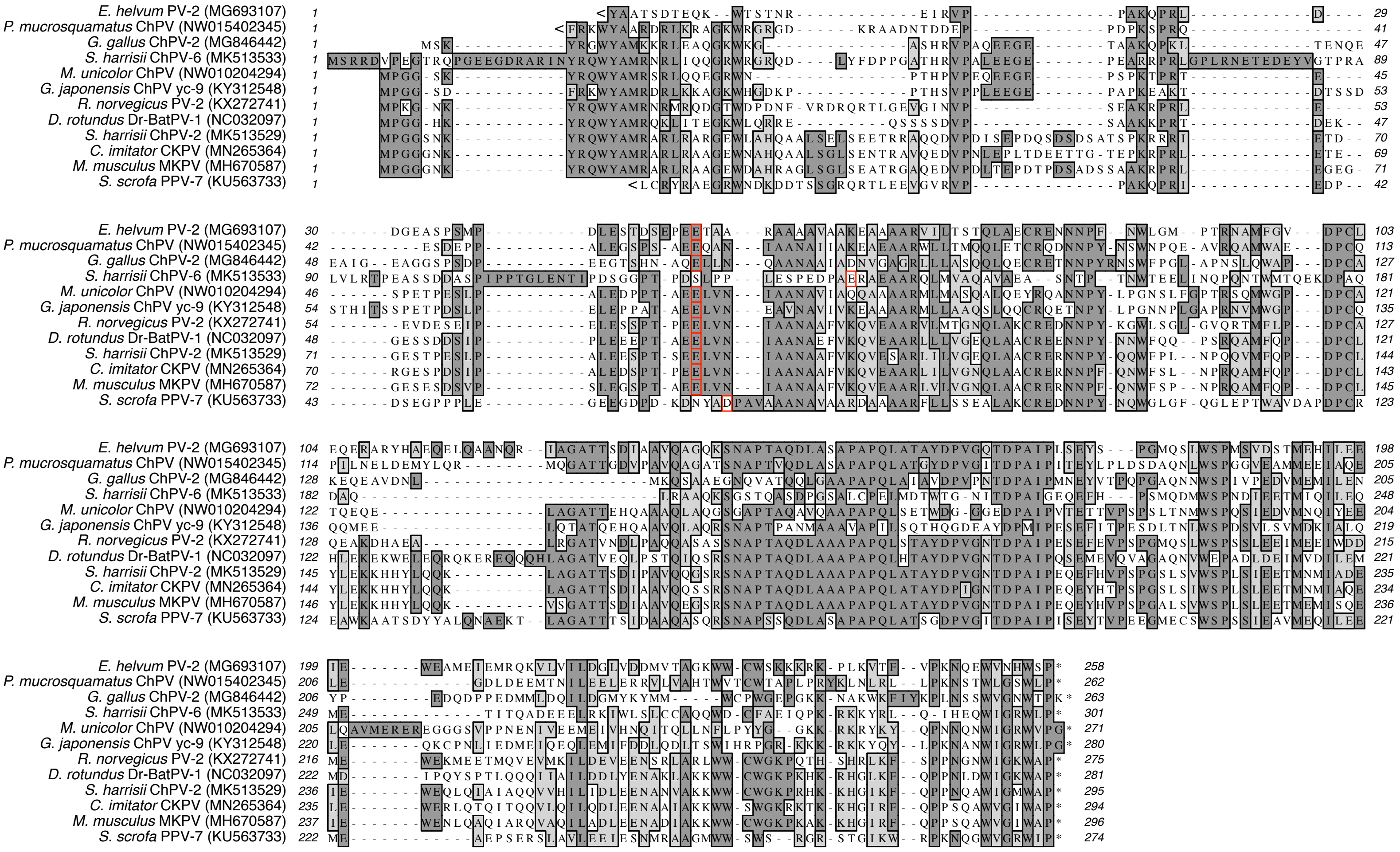

p15

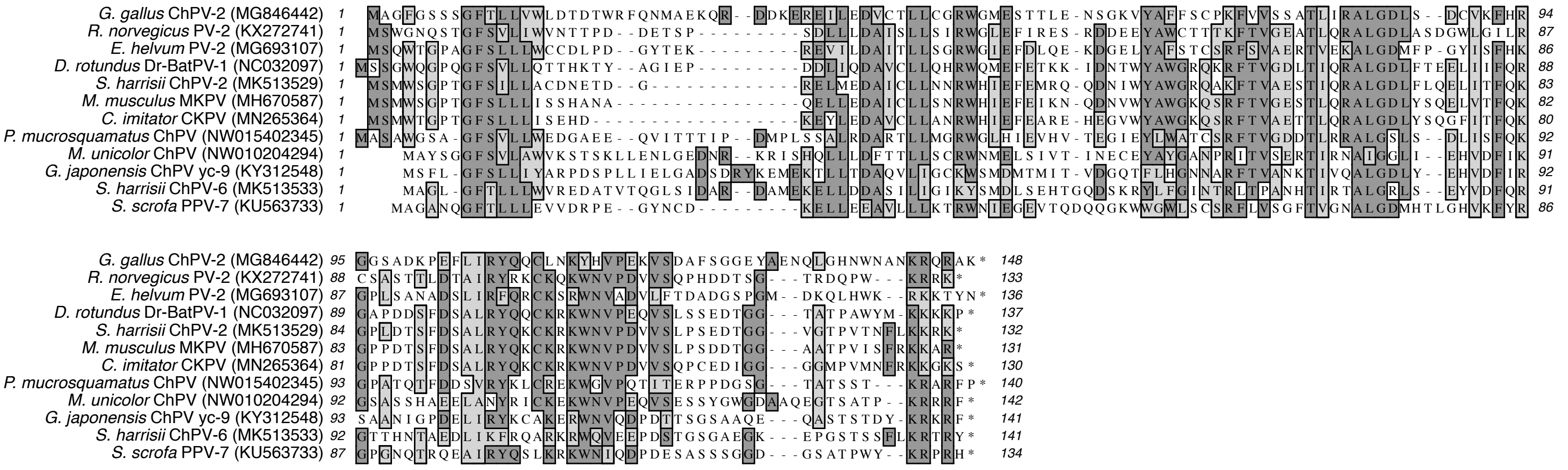

p10

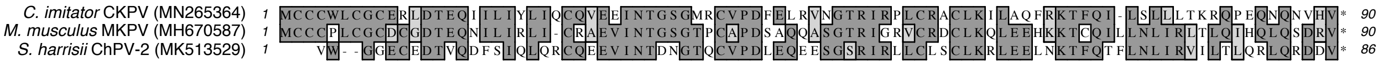

Figure S5A (cont.)

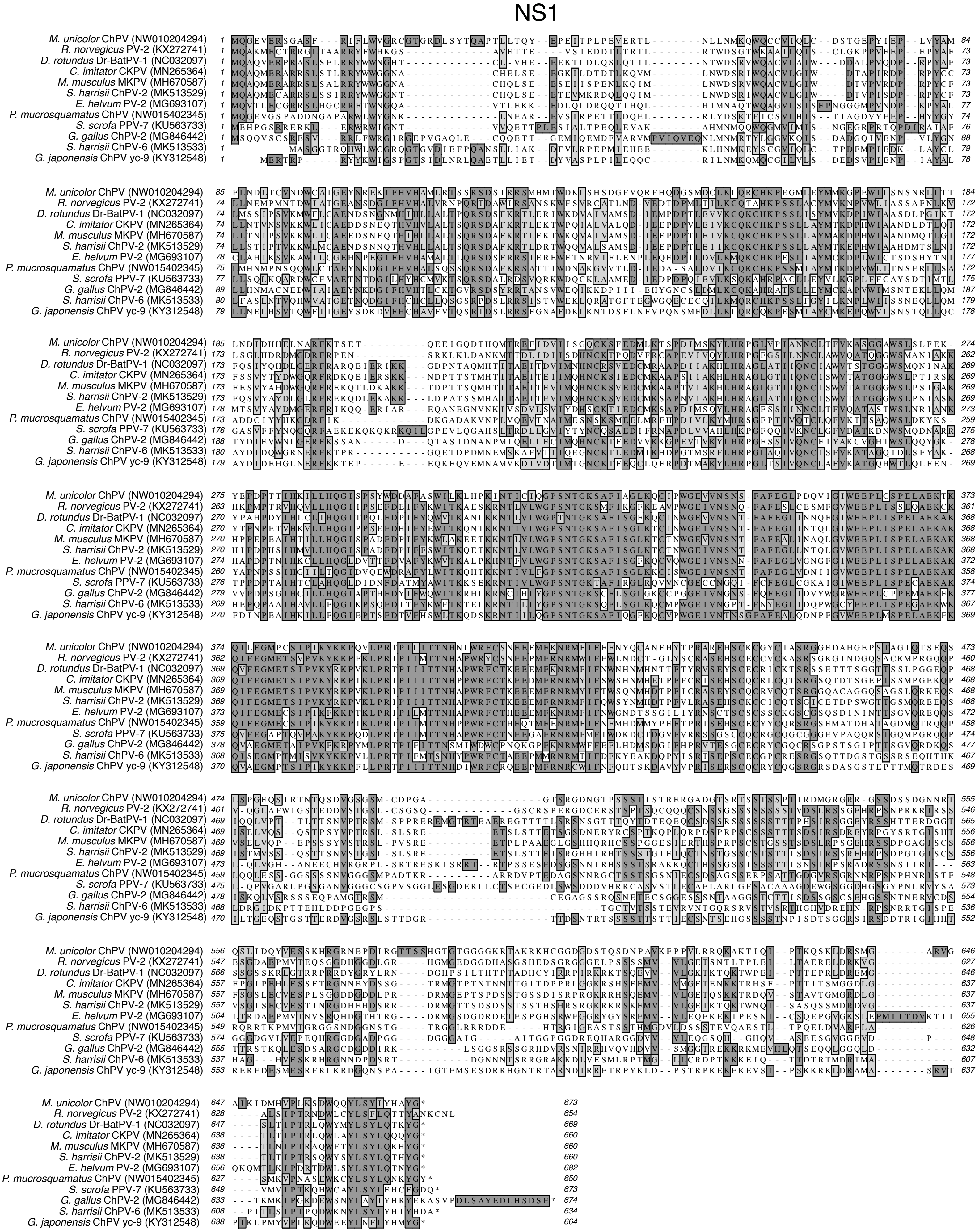

Figure S5B

VP

```
Mus musculus MKPV - MH670587
Splice score:   0.99                                                0.91
TCTCCAACTGCCGAAGGTAATTAAAAACCTTTTTTTATATCTTCTTACAGA.....ATGGTTCACTTATCTATCTTATTTACAGAAACACTATGGCTGAAGATGTCA
                 >                                   2289 bp          80% Pyr         <          M  A  E  D  V
```

```
Cebus imitator CKPV - KV391748
Splice score:   0.94                                                0.81
TCACCAACACCTGAAGGTACTTATCTTTTTATTTCTTTTATGCATGCAGAT.....ATGGCTTGCTTATCTCTCTTATTTACAGCAACAATATGGCTGAAGATATCA
                 >                                   2288 bp          87% Pyr         <          M  A  E  D  I
```

```
Sarcophilus harrisii ChPV 2 - MK513529
Splice score:   0.99                                                0.99
TCGCCTACATCAGAAGGTAATTATATTTACTCTTACTTTTCTTTCTAGATG.....ATGGTATTCTTATCTTTCCTATTTACAGACTCACTATGGCTGAAGATATAT
                 >                                   2290 bp          80% Pyr         <          M  A  E  D  I
```

```
Desmodus rotundus parvovirus strain DRA25 - NC_032097
Splice score:   1.00                                                0.89
GAACCCACAGCAGAAGGTAAGCTAACATTTGTATTATTTTTCATAGATGTC.....ATGGTATATGTATCTCTCCTACCTACAGACAAAATATGGCTGAAGATGTCA
                 >                                   2330 bp          80% Pyr         <          M  A  E  D  V
```

```
Ratus norvegicus parvovirus 2 isolate 9 - KX272741
Splice score:   0.89                                                0.46
TCTCCTACGCCTGAAGGTATTTATTCTTTTTGTTTTTCTCTCTTTTAGAAT.....GCTAATAAATGTAATCTTTGATTTACAGAATAAACATGGCAACTGATGTTT
                 >                                   2310 bp          67% Pyr         <          M  A  T  D  V
```

```
Eidolon helvum isolate BtPV/CMR/2014 - MG693107
Splice score:   0.89                                                0.95
GATTCTGAACCTGAAGGTATTTATTTTTATTTTATAGATGTCTCAGTGGAC.....CTGGTTATCTTACTTGTCTTATTTACAGACCAATTATGGCTGAAGATGTTT
                 >                                   2354 bp          80% Pyr         <          M  A  E  D  V
```

```
Protobothrops mucrosquamatus ChPV NW_015402345
Splice score:   0.90                                                0.85
AGTCCATCAGCAGAAGGTAATGAAACCTTTTCTTTTAGCATTTCAGGCTAT.....ATGGAAATGTTATTTGTCTTACTTGCAGAATAAATATGGCTACTGATCATG
                 >                                   2285 bp          80% Pyr         <          M  A  T  D  H
```

```
Sus scrofa PV7 isolate 42 - KU563733
Splice score:   0.98                                  0.66
AAGACAACTACGCAGGTGAGTGTGTAACTGCTTT.....CACTGGTGTGCTTATCTGTCCTATCTAGAGCATTGCTTCGGTGATCAATAAAGCGCCTGAGCTCAATCATGGCAGAACACATCACC
                >              2343 bp           73% Pyr       <                                            M  A  E  H  I  T
```

```
Mesitornis unicolor ChPV - NW_010204294
Splice score:   0.99                                                NS
CCTCCTACAGCTGAGGGTAAGCAATGGCGTATTCTGGCGGTTTTTC.....GCATGTACCTCTTAAATCTGATTGGCAGCAATACCTCAGCTACATCTACCACGCATATGGCTGAAGATTATACTA
                 >                          2307 bp          60% Pyr       <                                     M  A  E  D  Y  T
```

```
Grus japonicus PV isolate yc-9 - KY312548
Splice score: 0.59                                    0.41
CCAGCAACAGCTGAAGGTACAACATGTCTTTTCTGGGCTTTTCA.....ATTACCTATGTATGTTCCTTTAAAACAGGATTGGGAAGAATACCTGAATTTCCTGTATCATATGTATGGCTGAAGACGTCTCATA
                >                       2286 bp          60% Pyr      <                                          M  A  E  D  V  S  Y
```

```
Gallus gallus PV strain RS/BR/2S - MG846442
Splice score: 1.00                                                                          0.76
TCAGATCCTGAAGAAGGTACCAGTCATAATGCACAAGGTATGTCATTATTTTCTTATATTTCAGAG.....TTTATCTGCATACGAAGATCTACATTCAGATTCAGAATGACTGAAACAATAAAAT
                 >                           2398 bp                                         53% Pyr         <  M  T  E  T  I  K
```

```
Sarcophilus harrisii ChPV 6 - MK513533
Splice score:   0.99                                   0.98
CCTGAGGACCCAGCGGGTACGTATGGCAGGTCTAGGC.....CATAACACTATCTATTCCTACTCCACAGGACTGGAAGAATTACTTGTCTTACTTGTATCACATTTATCACGATGCCTGAATACACTAATTGTT
                >              2186 bp           80% Pyr       <                                                  M  P  E  Y  T  N  C
```

Figure S5B (cont.)

NS2

```
Mus musculus MKPV - MH670587
Splice score:   0.99                                                   0.58
TCTCCAACTGCCGAAGGTAATTAAAAACCTTTTTTTATATCTTCTTACAGA.....ATATGCATGATACACCATTTATTTGCAGAGCTAGTGAATATAGCTGCCAAT
 S  P  T  A  E  >                                  1622 bp          67% Pyr     <E  L  V  N  I  A  A  N

Cebus imitator CKPV - KV391748
Splice score:   0.94                                                   0.95
TCACCAACACCTGAAGGTACTTATCTTTTTATTTCTTTTATGCATGCAGAT.....ATATGCACGAAACACCTTTCTTTTGCAGAACTAGTGAACATAGCTGCCAAT
 S  P  T  P  E  >                                  1629 bp          73% Pyr     <E  L  V  N  I  A  A  N

Sarcophilus harrisii ChPV 2 - MK513529
Splice score:   0.99                                                   0.96
TCGCCTACATCAGAAGGTAATTATATTTACTCTTACTTTTCTTTCTAGATG.....ATATGCATGATACTCCTTTTGTTCTCAGAGCTAGTGAACATAGCTGCAAAT
 S  P  T  S  E  >                                  1623 bp          80% Pyr     <E  L  V  N  I  A  A  N

Desmodus rotundus parvovirus strain DRA25 - NC_032097
Splice score:   1.00                                                   0.51
GAACCCACAGCAGAAGGTAAGCTAACATTTGTATTATTTTTCATAGATGTC.....ACATGCATAACACCACTTTCACTTACAGAGTTAGTGAATATAGCTGCGAAT
 E  P  T  A  E  >                                  1636 bp          73% Pyr     <E  L  V  N  I  A  A  N

Ratus norvegicus parvovirus 2 isolate 9 - KX272741
Splice score:   0.89                                                   0.80
TCTCCTACGCCTGAAGGTATTTATTCTTTTTGTTTTTCTCTCTTTTAGAAT.....ATGATTGCACTGGTCTCTATTCTTGTAGAGCTAGTGAACATAGCTGCGAAT
 S  P  T  P  E  >                                  1612 bp          80% Pyr     <E  L  V  N  I  A  A  N

Eidolon helvum isolate BtPV/CMR/2014 - MG693107
Splice score:   0.89                                                   0.93
GATTCTGAACCTGAAGGTATTTATTTTTATTTTATAGATGTCTCAGTGGAC.....ACACTTCCTCTGGTATTTTGATTTATAGAAACAGCTGCACGAGCTGCAGCT
 D  S  E  P  E  >                                  1633 bp          67% Pyr     <E  T  A  A  R  A  A  A

Protobothrops mucrosquamatus ChPV NW_015402345
Splice score:   0.90                                                     NS
AGTCCATCAGCAGAAGGTAATGAAACCTTTTCTTTTAGCATTTCAGGCTAT.....ACATGATGTACCCTGAATTTACACCTAGAACAAGCGAACATAGCTGCGAAT
 S  P  S  A  E  >                                  1621 bp          60% Pyr     <E  Q  A  N  I  A  A  N

Sus scrofa PPV7 isolate 42 - KU563733
Splice score:   0.98                                                   0.93
AAAGACAACTACGCAGGTGAGTGTGTAACTGCTTTTGCTTTCGCTTTAGAT.....ACTGTACTGACGGTGTGTTCGTTCGCAGATCCAGCGGTAGCTGCTGCCAAT
 K  D  N  Y  A  >                                  1663 bp          60% Pyr     <D  P  A  V  A  A  A  N

Mesitornis unicolor ChPV - NW_010204294
Splice score:   0.99                                                     NS
CCTCCTACAGCTGAGGGTAAGCAATGGCGTATTCTGGCGGTTTTTCGGTGC.....AATGCGCAAACGAACATTATACTCCTAGAGCTCGTGAACATAGCTGCAAAT
 P  P  T  A  E  >                                  1637 bp          53% Pyr     <E  L  V  N  I  A  A  N

Grus japonicus PV isolate yc-9 - KY312548
Splice score:   0.59                                                   0.50
CCAGCAACAGCTGAAGGTACAACATGTCTTTTCTGGGCTTTTCATTATTAA.....CTTCAAAAGATGCTGTATATGTTCCCAGAATTAGTGAACGAAGCTGTCAAT
 P  A  T  A  E  >                                  1640 bp          60% Pyr     <E  L  V  N  E  A  V  N

Gallus gallus PV strain RS/BR/2S - MG846442
Splice score:   1.00                                                   0.94
TCAGATCCTGAAGAAGGTACCAGTCATAATGCACAAGGTATGTCATTATTT.....ATATGTCTGATGGTATTTTTCATCCTAGAGTTACTGAATCAGGCTGCGAAT
 S  D  P  E  E  >                                  1685 bp          67% Pyr     <E  L  L  N  Q  A  A  N

Sarcophilus harrisii ChPV 6 - MK513533
Splice score:   0.99                                                   0.49
CCTGAGGACCCAGCGGGTACGTATGGCAGGTCTAGGCTTTACCCTATTGCT.....AACCGAGCTGTGAGTGCGGTCCTTGCAGAGAGAGCAGAGGCAGCCAGACAA
 P  E  D  P  A  >                                  1666 bp          53% Pyr     <E  R  A  E  A  A  R  Q
```

Figure S5B (cont.)

NS1 or p15

```
Mus musculus MKPV - MH670587
Splice score:   0.99                                            0.98
CTAGAAGGAGAAGGAGGTGAGTCAGAATCGGA.....ATTAAAAACCTTTTTTTATATCTTCTTACAGATGTCTATGTGGTCA
                >              88/86 bp              87% Pyr      <
                                                              p15>   M  S  M  W  S
```

```
Cebus imitator CKPV - KV391748
Splice score:   1.00                                            0.55
CTAGAAACCGAAAGAGGTGAGTCGCCAGACTC.....ACTTATCTTTTTATTTCTTTTATGCATGCAGATGTCTATGTGGACG
                >                84 bp                73% Pyr      <
                                                          p15> (+2) <M  S  M  W  T
```

```
Sarcophilus harrisii ChPV 2 - MK513529
Splice score:   0.99                                            0.99
CGTATAGAGACAGACGGTGAGTCAACTCCTGA.....TAATTATATTTACTCTTACTTTTCTTTCTAGATGTCAATGTGGTCT
                >                 86 bp               93% Pyr      <
                                                              p15>   M  S  M  W  S
```

```
Desmodus rotundus parvovirus strain DRA25 - NC_032097
Splice score:   0.99                                            1.00
CGAACAGACGAAAAGGGTGAGTCTTCAGACGA.....GGTAAGCTAACATTTGTATTATTTTTCATAGATGTCTAGCGGCTGG
                >                84 bp                80% Pyr      <
                                                              p15>   M  S  S  G  W
```

```
Ratus norvegicus parvovirus 2 isolate 9 - KX272741
Splice score:   0.84                                           0.99
GGGACACAGTTTCCTGGTAGGCGTCAGTTGCG.....ATTTATTCTTTTTGTTTTTCTCTCTTTTAGAATGTCCTGGGGAAAT
                >                262 bp               93% Pyr     <
                                                         p15> (+71) <M  S  W  G  N
```

```
Eidolon helvum isolate BtPV/CMR/2014 - MG693107
Splice score:   0.96                                           0.99
CTGCTAAACAACCAAGGTTAGACGACGGGGAG.....GAACCTGAAGGTATTTATTTTTATTTTATAGATGTCTCAGTGGACG
                >                86 bp                80% Pyr      <
                                                              p15>   M  S  Q  W  T
```

```
Protobothrops mucrosquamatus ChPV NW_015402345
Splice score:   0.95                                         0.97
   <CTTTCGGAAATGGTACGCAGCGCGTGAT.....AATGAAACCTTTTCTTTTAGCATTTCAGGCTATGGCGTCTGCATGG
                >                188 bp            80% Pyr      <
                                                        p15> (+10)  <M  A  S  A  W
```

```
Sus scrofa PV7 isolate 42 - KU563733
Splice score:   0.67                                            1.00
GCGGACACTCGAGGAGGTAGGGGTCCGGGTCC.....GAGTGTGTAACTGCTTTTGCTTTCGCTTTAGATGGCTGGCGCGAATG
                >                145 bp                80% Pyr     <
                                                              p15>   M  A  G  A  N
```

```
Mesitornis unicolor ChPV - NW_010204294
Splice score:   0.95                               0.46
AGTCTGCTTCTATCAGGTAATGCCT.....CCCGCTCTCGAAGATCCTCCTACAGCTGAGGGTAAGCAATGGCGTATTCTGGCG
                >         187 bp          67% Pyr     <
                                                              p15>   M  A  Y  S  G
```

```
Grus japonicus PV isolate yc-9 - KY312548
Splice score:    0.99                                        0.96
TAGATTAAAAAAAGCTGGTAAGTGGCATGGAGATAAACCTCCAACTCATTCTGTTCCTCCATTAGAAGAAGGCGA.....CAACAGCTGAAGGTACAACATGTCTTTTCTGG
                 >               48 bp             80% Pyr      <
                                                                                             p15>   M  S  F  L
```

```
Gallus gallus PV strain RS/BR/2S - MG846442
Splice score:    0.99                                          0.96
CTAGGGTATAAGTATGAGTAAGTACA.....ATGCACAAGGTATGTCATTATTTTCTTATATTTCAGAGTATCTATGGCGGGCTTTGGAT
                 >               231 bp              80% Pyr      <
                                                                    p15>   M  A  G  F  G
```

```
Sarcophilus harrisii ChPV 6 - MK513533
Splice score:   0.96                                    0.40
CCTGAGGACCCAGCGGGTACGAGACAGCC.....GGGACCCCAAGAGCGCTCGTCCTTAGACCCCAGAGGCCT.....CCCAGCGGGTACGTATGGCAGGTCTAGGCT
                >            253 bp           53% Pyr      <                          p15>   M  A  G  L  G
```
