# Supplementary material for: Murine and related chapparvoviruses are nephro-tropic and produce novel accessory proteins in infected kidneys
Source: PLoS Pathog. 2020 Jan 23;16(1):e1008262. doi: 10.1371/journal.ppat.1008262 (PMC6999912; doi:10.1371/journal.ppat.1008262)
Supplement: S1 Table — (A) MKPV PCR primers used in this study and (B) product sizes from the MKPV genome or MKPV transcript 1–4 cDNAs. (PDF) [file ppat.1008262.s006.pdf]

Table S1A

PCR primers used in this study

MKPV primers	Sequence (5'–3')	MKPV bases (MH670587)
869	GCAACACAACTGTAAATCGG	1458–1477
870	TGGATAATCGTGCCTAAACC	1534–1553
889	GGGCAGAGAGACTACGTCGGGC	832–853
890	CACGCAGTGCGCGCACATGC	75–94
891	GGTCAGATACAGAACAGGAGG	3991–4011
893	ACATGTTCTGTACGACTGGG	4372–4391
900	ATTCACTAGCTCTTCGGCAG	478–486:2109–2119
902	TTAGAGAGCAGGCAGATAGC	602–621
904	ATTGAATTCATATGCCGGGCGGAGGTA	214–234
905	TGTGCGGCCGCTTAAGGTGCCCAGATCCC	2711–2728
932	AGGCACTACCGCTTTCACTG	3061–3080
933	GAAAGCTTTGTAATCTCCTGTG	3163–3184
934	CACCTCACAGAGATCAGATGC	1119–1139
935	TATGCATCTGTGAGGTGGTC	1407–1426
940	AGCCGCATCTATGCACCAAC	3911–3930
947	GACGTGCCAGATCTTACTGAACC	348–370
948	GTGACATCTTCAGCCATAGTG	2779–2799
955	CTGCCAGTTTCGTAGCAGTC	131–150
**DrPV-1 primers**		**DrPV-1 bases (NC032097)**
Chap-DRPv-fwd	GCAAGCGCAAGTGGAACG	654:671
Chap-DRPv-rev	GACCATCCTCCTCCTGTAG	1418:1436
**RACE primers**		
SMARTer CDS primer	CGGGGTACGATGAGACACCATTTTTTTTTTTTTTTTTTTTTTVN	
Template Switch oligo	AAGCAGTGGTATCAACGCAGAGTACAGGG	
5' RACE primer	TTAAGCAGTGGTATCAACGCAGAGTACAG	
3' RACE primer	CGGGGTACGATGAGACACCA	

Table S1B

MKPV PCR product sizes (bp)

		3' PRIMER		
**TEMPLATE**	**5' PRIMER**	**902**	**905**	**933**
**genome**	**890**	547	2654	3184
**genome**	**955**	491	2598	3054
**Transcript 1**		491	2598	3054
**Transcript 2**		403	2510	2966
**Transcript 3**		–	1409	1865
**Transcript 4**		–	–	765
**genome**	**904**	408	2515	2971
**Transcript 1**		408	2515	2971
**Transcript 2**		320	2427	2883
**Transcript 3**		–	1326	1782
**Transcript 4**		–	–	682
**genome**	**947**	274	2381	2837
**Transcript 1**		274	2381	2837
**Transcript 2**		186	2293	2749
**Transcript 3**		–	1192	1648
**Transcript 4**		–	–	548
