# Supplementary material for: Murine and related chapparvoviruses are nephro-tropic and produce novel accessory proteins in infected kidneys
Source: PLoS Pathog. 2020 Jan 23;16(1):e1008262. doi: 10.1371/journal.ppat.1008262 (PMC6999912; doi:10.1371/journal.ppat.1008262)
Supplement: S2 Table — (PDF) [file ppat.1008262.s007.pdf]

Table S2

Summary of significant MKPV peptides in independent LC-MS/MS dataset PXD014938

Porotein Accession	-10lgP	Coverage (%)	Area Sample 1	#Peptides	#Unique	#Spec Sample 1	PTM	Avg. Mass
MKPV_p15	145.69	63	1.50E+09	12	12	34		14683
MKPV_NS1	152.56	28	1.91E+08	14	14	22		73471
MKPV_VP1	53.89	10	1.59E+07	4	4	4	Deamidation (NQ)	57170
MKPV_p10	38.41	15	2.20E+07	2	2	3		9815

Protein Accession	Peptide	Start–End	Unique	-10lgP	Mass	Length	ppm	m/z	z	RT	Area Sample 1	Fraction	Scan	Source File	#Feature	#Feature Sample 1	Start	End	PTM	AScore
MKPV_p15	R.WHIEFEIK.N	36–43	Y	38.56	1100.5654	8	1.4	367.8629	3	74.99	2.47E+07	1	31328	Infected_01.raw	1	1	36	43		
MKPV_p15	K.NQDNVWYAWGK.Q	44–54	Y	48.61	1379.6259	11	0.4	690.8205	2	72.58	3.76E+07	1	29917	Infected_01.raw	1	1	44	54		
MKPV_p15	R.FTVGESTLQR.A	58–67	Y	41.61	1136.5825	10	-0.8	569.2981	2	49.57	5.95E+08	1	17244	Infected_01.raw	1	1	58	67		
MKPV_p15	R.ALGDLYSQELVTF.Q	68–80	Y	20.05	1454.7292	13	-1.1	728.3711	2	96.71	1.87E+06	1	41486	Infected_01.raw	1	1	68	80		
MKPV_p15	R.ALGDLYSQELVTFQK.G	68–82	Y	53.78	1710.8828	15	1.6	856.4501	2	80.66	2.28E+08	1	33880	Infected_01.raw	3	3	68	82		
MKPV_p15	K.GPPDTSFDSALR.Y	83–94	Y	57.38	1261.5939	12	-2.5	631.8026	2	55.06	4.86E+08	1	20336	Infected_01.raw	1	1	83	94		
MKPV_p15	R.KWNVPDVVSLPSDDTGGAATPVISF.R	101–125	Y	17.64	2571.2856	25	3.4	1286.6544	2	96.06	9.41E+05	1	41124	Infected_01.raw	1	1	101	125		
MKPV_p15	R.KWNVPDVVSLPSDDTGGAATPVISFR.K	101–126	Y	42.49	2727.3867	26	0.5	910.1366	3	86.37	6.13E+07	1	36772	Infected_01.raw	1	1	101	126		
MKPV_p15	K.WNVPDVVSLPSDDTGGAATPVISFR.K	102–126	Y	35.54	2599.2917	25	-1	867.437	3	94.02	5.11E+07	1	40317	Infected_01.raw	2	2	102	126		
MKPV_p15	N.VPDVVSLPSDDTGGAATPVISFR.K	104–126	Y	21.74	2299.1694	23	-0.6	1150.5913	2	82.81	2.41E+06	1	35047	Infected_01.raw	1	1	104	126		
MKPV_p15	S.LPSDDTGGAATPVISFR.K	110–126	Y	35.6	1702.8525	17	-0.9	852.4327	2	67.62	7.23E+06	1	27168	Infected_01.raw	1	1	110	126		
MKPV_p15	S.DDTGGAATPVISFR.K	113–126	Y	41.3	1405.6837	14	-1.6	703.848	2	64.49	7.43E+06	1	25529	Infected_01.raw	1	1	113	126		
MKPV_NS1	K.QIMLNWDSR.V	42–50	Y	25.28	1161.5601	9	-1.5	581.7864	2	67.44	5.21E+06	1	27089	Infected_01.raw	1	1	42	50		
MKPV_NS1	K.DPHWIAANDMQTLGIFESVYAHDWGQR.F	158–184	Y	45.71	3156.4512	27	0.2	790.1202	4	100.93	4.17E+06	1	43216	Infected_01.raw	2	2	158	184		
MKPV_NS1	K.HPPEPEAIHTILLHQGISPADFDPIFYK.W	270–297	Y	46.66	3181.6235	28	-0.8	796.4125	4	87.47	3.36E+07	1	37445	Infected_01.raw	1	1	270	297		
MKPV_NS1	H.QGISPADFDPIFYK.W	284–297	Y	29.57	1596.7823	14	0.9	799.3992	2	88.01	3.13E+06	1	37637	Infected_01.raw	1	1	284	297		
MKPV_NS1	K.NTLVLWGPSNTGK.S	307–319	Y	50.48	1385.7303	13	-3.9	693.8698	2	68.87	1.10E+07	1	27859	Infected_01.raw	1	1	307	319		
MKPV_NS1	K.SAFISGLK.T	320–327	Y	30.68	821.4647	8	-0.8	411.7393	2	54.48	1.43E+07	1	20107	Infected_01.raw	1	1	320	327		
MKPV_NS1	K.QIFEGMETSIPVK.Y	369–381	Y	49.41	1477.7487	13	-1.3	739.8806	2	71.09	1.24E+07	1	29127	Infected_01.raw	1	1	369	381		
MKPV_NS1	R.IPIIITTNH.A	391–399	Y	30.37	1020.5968	9	-0.3	511.3055	2	61.05	4.36E+06	1	23647	Infected_01.raw	1	1	391	399		
MKPV_NS1	R.IPIIITTNHAPWR.F	391–403	Y	44.49	1530.8671	13	2.3	511.2975	3	69.4	1.18E+07	1	28127	Infected_01.raw	2	2	391	403		
MKPV_NS1	R.KEQSVSELVQPEPSSSY.V	465–481	Y	23.98	1892.9003	17	-3.2	947.4543	2	57.69	7.75E+06	1	21777	Infected_01.raw	1	1	465	481		
MKPV_NS1	R.EETPLPAAEGLGSHHQR.H	492–508	Y	57.59	1827.8864	17	-1.5	457.9782	4	41.7	3.21E+07	1	13023	Infected_01.raw	2	2	492	508		
MKPV_NS1	R.MGEPTSPDSSTGSSDISRPR.G	582–601	Y	22.04	2062.9226	20	-2.1	688.6467	3	36.37	1.09E+06	1	10183	Infected_01.raw	1	1	582	601		
MKPV_NS1	R.DQVSTAVTGMGR.D	623–634	Y	44.84	1220.5819	12	0.6	611.2986	2	46.18	2.45E+07	1	15480	Infected_01.raw	1	1	623	634		
MKPV_NS1	R.DLGTLNIPTR.A	635–644	Y	31.61	1098.6033	10	-0.6	550.3086	2	62.65	2.52E+07	1	24473	Infected_01.raw	1	1	635	644		
MKPV_VP1	K.NDYTIPNLLDMPIVPMQWFWQEIQK.S	287–311	Y	22.49	3118.5295	25	-1.2	1040.5159	3	134.06	3.74E+05	1	56548	Infected_01.raw	1	1	287	311		
MKPV_VP1	K.SIAEVPDVK.K	312–320	Y	35.45	956.5178	9	-0.9	479.2657	2	47.33	7.63E+06	1	16005	Infected_01.raw	1	1	312	320		
MKPV_VP1	K.ATMHPYEQAASGMY.L	456–469	Y	15.06	1555.6436	14	-2.9	778.8268	2	53.71	3.53E+06	1	19606	Infected_01.raw	1	1	456	469		
MKPV_VP1	K.ATMHPYEQAASGMYLN(+.98)HK.E	456–473	Y	21.55	2048.9084	18	-2.8	683.9749	3	50.6	4.34E+06	1	17899	Infected_01.raw	1	1	456	473	Deamidation (NQ)	N16:Deamidation (NQ):8.51
MKPV_p10	R.LTLQIHQLQSDR.V	77–88	Y	25.52	1450.7892	12	-0.4	484.6035	3	55.37	6.09E+06	1	20525	Infected_01.raw	1	1	77	88		
MKPV_p10	R.LTLQIHQLQSDRV	77–89	Y	24.92	1549.8577	13	-0.3	517.6263	3	61.41	1.59E+07	1	23827	Infected_01.raw	2	2	77	89		
