# Supplementary material for: Murine and related chapparvoviruses are nephro-tropic and produce novel accessory proteins in infected kidneys
Source: PLoS Pathog. 2020 Jan 23;16(1):e1008262. doi: 10.1371/journal.ppat.1008262 (PMC6999912; doi:10.1371/journal.ppat.1008262)
Supplement: S3 Table — (PDF) [file ppat.1008262.s008.pdf]

Table S3

Summary of significant MKPV peptides in independent LC-MS/MS dataset PXD010540

Sequence	Unique	Start–End	-10lgP	Mass expected	Mass observed	Mass Error (ppm)	Score	Expectation Value
**MKPV p15; 130 residues**								
K.NQDNVWYAWGK.Q	Y	44–54	3.74	1379.6280	1379.6258	1.59	37	1.800E-04
R.FTVGESTLQR.A	Y	58–67	5.66	1136.5816	1136.5826	-0.88	57	2.200E-06
R.ALGDLYSQELVTFQK.G	Y	68–82	4.11	1710.8825	1710.8828	-0.18	41	7.800E-05
K.GPPDTSFDSALR.Y	Y	83–94	8.07	1261.5964	1261.5939	1.98	81	8.600E-09
K.WNVPDVVSLPSDDTGGAATPVISFR.K	Y	102–126	5.00	2599.2940	2599.2919	0.81	50	1.000E-05
**MKPV NS1; 659 residues**								
K.NTLVLWGPSNTGK.S	Y	307–319	2.85	1385.7315	1385.7303	0.87	28	1.400E-03
R.KEQSVSELVQPEPSSSYVSTR.S	Y	465–485	3.96	2336.1511	2336.1496	0.64	40	1.100E-04
R.EETPLPAAEGLGSHHQR.H	Y	492–508	4.82	1827.8895	1827.8864	1.70	48	1.500E-05
R.DQVSTAVTGMGR.D	Y	623–634	5.28	1220.5815	1220.5820	-0.41	53	5.200E-06
R.DLGTLNIPTR.A	Y	635–644	4.82	1098.6006	1098.6033	-2.46	48	1.500E-05
**MKPV VP; 496 residues**								
K.ATMHPYEQAASGMYLNHK.E	Y	456–473	2.49	2047.9182	2047.9244	-3.03	25	3.200E-03
**MKPV p10; 89 residues**								
R.LTLQIHQLQSDRV	Y	77–89	7.89	1549.8595	1549.8574	1.35	79	1.300E-08
