# Supplementary material for: Murine and related chapparvoviruses are nephro-tropic and produce novel accessory proteins in infected kidneys
Source: PLoS Pathog. 2020 Jan 23;16(1):e1008262. doi: 10.1371/journal.ppat.1008262 (PMC6999912; doi:10.1371/journal.ppat.1008262)
Supplement: S4 Table — (PDF) [file ppat.1008262.s009.pdf]

Table S4 Summary of MKPV splicing in dataset GSE117710

donor:acceptor nt	splice donor*	splice acceptor*	Read Counts Mouse 1	Read Counts Mouse 2	Read Proportion Mouse 1	Read Proportion Mouse 2	notes
487:2775	GCCGAAGGTAATT	TATCTATCTTATTTACAGAAA	5038	296	3.8E-01	4.9E-01	VP, p10 CDS
487:2108	GCCGAAGGTAATT	TACACCATTTATTTGCAGAGC	2962	110	2.2E-01	1.8E-01	NS2, p10 CDS
433:520	AAGGAGGTGAGT	TTTTATATCTTCTTACAGATG	2589	52	1.9E-01	8.7E-02	p15, NS1, NP ± p10 CDS
retain 487–520	n/a	n/a	1835	43	1.4E-01	7.2E-02	p15, NS1, NP, p10 CDS
433:2775	AAGGAGGTGAGT	TATCTATCTTATTTACAGAAA	347	43	2.6E-02	7.2E-02	VP, p10 CDS
232:390	GCGGAGGTAATA	ATACGCCTGACTCTGCAGATT	243	5	5.0E-03	8.4E-03	
2588:2775	CGACAGGTAGCT	TATCTATCTTATTTACAGAAA	143	35	1.1E-02	5.9E-02	alters C-terminus of NS1, NS2 or NP?
283:390	CAGCAGGCGAGT	ATACGCCTGACTCTGCAGATT	77	0	5.7E-03	0	
667:2775	TGTCTGGTATGC	TATCTATCTTATTTACAGAAA	69	8	5.1E-03	1.3E-02	VP1, p10 CDS
433:2108	AAGGAGGTGAGT	TACACCATTTATTTGCAGAGC	67	5	1.8E-02	8.4E-03	removes 18 aa from exon 1 of NS2
2189:2775	CAGCTGGCAGCT	TATCTATCTTATTTACAGAAA	19	0	1.4E-03	0	
433:745	AAGGAGGTGAGT	GGTGATCTCTATTCACAGGAG	13	0	9.7E-04	0	
others	n/a	n/a	<5	<5	7.5E-05	1.7E-03	

* exon = black, intron = red; n/a = not applicable
