# Supplementary material for: Murine and related chapparvoviruses are nephro-tropic and produce novel accessory proteins in infected kidneys
Source: PLoS Pathog. 2020 Jan 23;16(1):e1008262. doi: 10.1371/journal.ppat.1008262 (PMC6999912; doi:10.1371/journal.ppat.1008262)
Supplement: S5 Table — (PDF) [file ppat.1008262.s010.pdf]

# Table S5

Summary of ISH in multiple tissues.

| Animal description (1) | | | | | | |
|---|---|---|---|---|---|---|
| **MSKCC-WCM Accession #** | 16-1653-1 | | | 15-3577-1 | | |
| **Sex** | Female | | | Female | | |
| **Age** | Unknown (adult) | | | 12 months | | |
| **Strain** | NSG | | | NSG | | |
| **Cause of death** | Inclusion body nephropathy caused by MKPV | | | Inclusion body nephropathy caused by MKPV | | |
| **Previously reported MKPV testing (1)** | | | | | | |
| **MKPV PCR on FFPE kidney tissue** | Positive | | | Positive | | |
| **MKPV ISH on FFPE kidney tissue** | Strongly positive, tubular cells, multifocal | | | NP | | |
| **ISH results** | **MKPV ISH** | **Mouse Ppib ISH (2)** | **Dapb ISH (3)** | **MKPV ISH** | **Mouse Ppib ISH** | **Dapb ISH** |
| **Heart** | Negative | NP | NP | Negative | Positive | NP |
| **Lungs** | Negative | NP | NP | Negative | Positive | NP |
| **Thymus** | U | NP | NP | Negative | Positive | NP |
| **Kidneys** | U (4) | NP | NP | Strongly positive, tubular cells, multifocal | NP | NP |
| **Liver** | Negative | NP | NP | Negative | NP | NP |
| **Gallbladder** | Negative | NP | NP | Negative | NP | NP |
| **Stomach** | Negative | NP | NP | Negative | NP | NP |
| **Duodenum** | Negative | NP | NP | Negative | NP | NP |
| **Jejunum** | Negative | NP | NP | Negative | NP | NP |
| **Ileum** | Negative | NP | NP | Negative | NP | NP |
| **Cecum** | Mildly positive, mucosal epithelium and lamina propria, multifocal | NP | NP | Negative | NP | NP |
| **Colon** | Negative | NP | NP | U | NP | NP |
| **Salivary glands** | Negative | NP | NP | Negative | NP | NP |
| **Uterus** | Negative | Positive | NP | Negative | NP | NP |
| **Urinary bladder** | Mildly positive, urothelium multifocal; Strongly positive, casts in lumen | Positive | NP | Mildly positive, urothelium multifocal; Strongly positive, casts in lumen | NP | NP |
| **Pancreas** | Negative | NP | NP | Negative | NP | NP |
| **Adrenals** | Negative | Positive | Negative | U | NP | NP |
| **Ovaries** | Negative | Positive | Negative | Negative | NP | NP |
| **Oviducts** | Negative | Positive | Negative | Negative | NP | NP |
| **Trachea** | Negative | Positive | Negative | Negative | Positive | NP |
| **Esophagus** | Negative | Positive | Negative | Negative | Positive | NP |
| **Thyroid** | Negative | Positive | Negative | Negative | NP | NP |
| **Skin (trunk)** | Negative | Positive | Negative | U | NP | NP |
| **Skeletal muscles** | Negative | NP | NP | Negative | NP | NP |
| **Bones (femur, tibia, sternum, vertebrae, skull)** | Not interpreted (5) | NP | NP | Not interpreted (5) | NP | NP |
| **Bone marrow (femur, tibia, sternum, vertebrae)** | Not interpreted (5) | NP | NP | Not interpreted (5) | NP | NP |
| **Stifle joint** | Not interpreted (5) | NP | NP | Not interpreted (5) | NP | NP |
| **Nerves (hind limb, spine)** | Not interpreted (5) | NP | NP | Not interpreted (5) | NP | NP |
| **Spinal cord** | Not interpreted (5) | NP | NP | Not interpreted (5) | NP | NP |
| **Oral mucosa** | Not interpreted (5) | NP | NP | Not interpreted (5) | Negative (5) | Negative |
| **Teeth** | Not interpreted (5) | NP | NP | Not interpreted (5) | Negative (5) | Negative |
| **Nasal mucosa** | Not interpreted (5) | NP | NP | Not interpreted (5) | Negative (5) | Negative |
| **Eyes** | Not interpreted (5) | NP | NP | Not interpreted (5) | Negative (5) | Negative |
| **Harderian gland** | Not interpreted (5) | NP | NP | Not interpreted (5) | Negative (5) | Negative |
| **Pituitary** | Not interpreted (5) | Negative (5) | Negative | Not interpreted (5) | NP | NP |
| **Brain** | Not interpreted (5) | Negative (5) | Negative | Not interpreted (5) | Negative (5) | Negative |
| **Ears** | Not interpreted (5) | Negative (5) | Negative | Not interpreted (5) | NP | NP |

**Notes:**
1. As described in reference 9.
2. Positive control probe.
3. Negative control probe.
4. Tissue exhausted by previous ISH staining; kidney sample from this mouse was previously found to be ISH positive as described in reference 9.
5. Due to inadequate RNA preservation caused by formic acid decalcification, as shown by negative Ppib results on all decalcified tissues tested.

NSG, NOD.Cg-*Prkdc*$^{scid}$ *Il2rg*$^{tm1Wjl}$/SzJ; FFPE, formalin-fixed paraffin-embedded; NP, not performed; U, sample unavailable.
