# Supplementary material for: Murine and related chapparvoviruses are nephro-tropic and produce novel accessory proteins in infected kidneys
Source: PLoS Pathog. 2020 Jan 23;16(1):e1008262. doi: 10.1371/journal.ppat.1008262 (PMC6999912; doi:10.1371/journal.ppat.1008262)
Supplement: S2 Fig — (A) Comparison of the amino acid sequences of the ORFs encoded by MKPV/MuCPV accessions MH670587, MH670588 and MF175078, in that order. (B) A variant telomere structure created by a single-base insertion in one of the right TR repeats of a sub-strain of CI-MKPV (indicated by “▲” in Fig 1A). (PDF) [file ppat.1008262.s002.pdf]

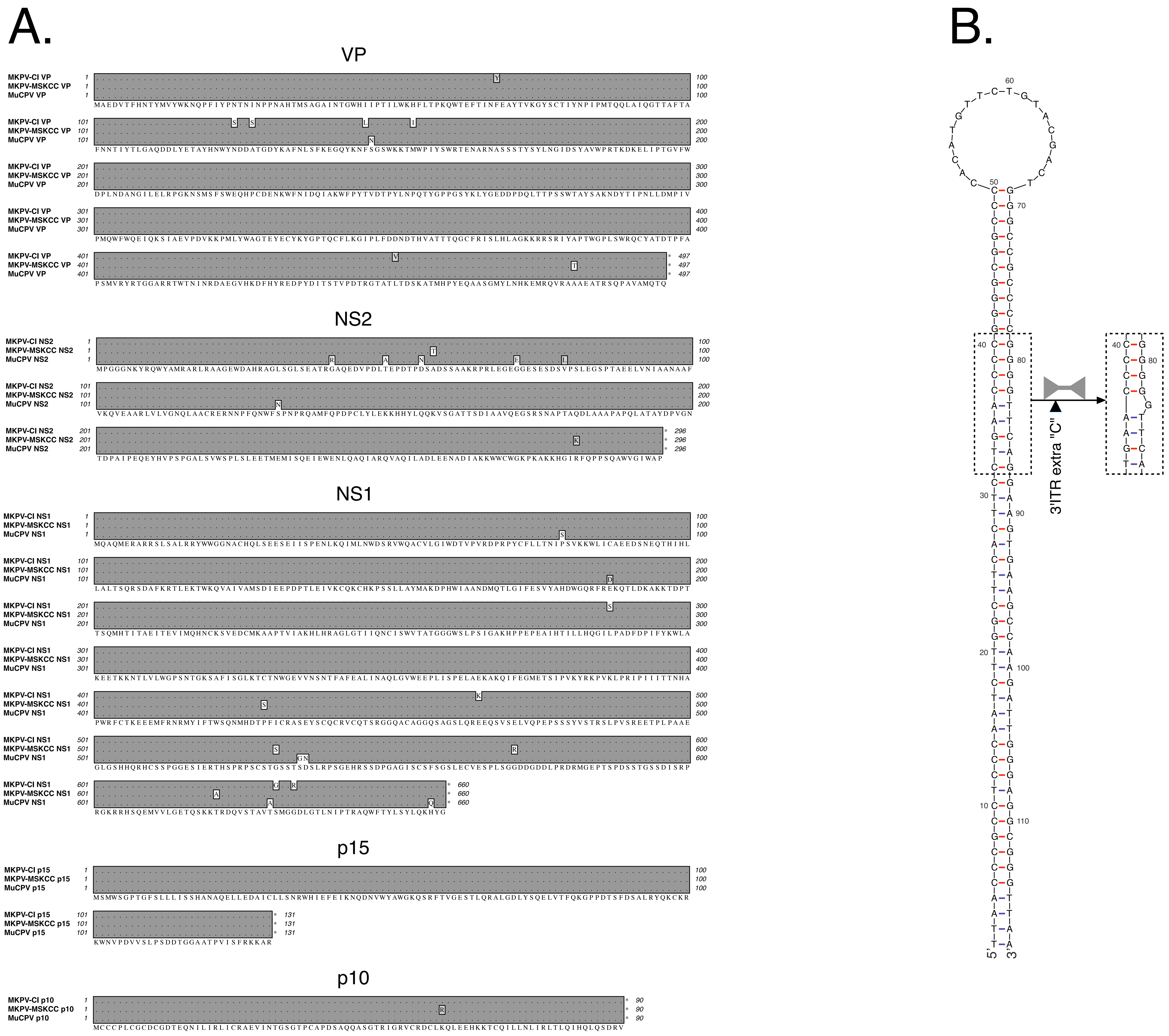
A.
VP
NS2
NS1
p15
p10
B.
3'ITR extra "C"
5'
3'
